# mRNA vaccine designs for optimal adjuvanticity and delivery

**DOI:** 10.1080/15476286.2024.2333123

**Published:** 2024-03-26

**Authors:** Yuki Mochida, Satoshi Uchida

**Affiliations:** a Department of Advanced Nanomedical Engineering, Medical Research Institute, Tokyo Medical and Dental University (TMDU), Tokyo, Japan; b Innovation Center of NanoMedicine (iCONM), Kawasaki Institute of Industrial Promotion, Kawasaki, Japan

**Keywords:** mRNA vaccine, adjuvant, innate immunity, mRNA delivery, lipid nanoparticle, polyplex, antigen-presenting cell, lymphoid organ

## Abstract

Adjuvanticity and delivery are crucial facets of mRNA vaccine design. In modern mRNA vaccines, adjuvant functions are integrated into mRNA vaccine nanoparticles, allowing the co-delivery of antigen mRNA and adjuvants in a unified, all-in-one formulation. In this formulation, many mRNA vaccines utilize the immunostimulating properties of mRNA and vaccine carrier components, including lipids and polymers, as adjuvants. However, careful design is necessary, as excessive adjuvanticity and activation of improper innate immune signalling can conversely hinder vaccination efficacy and trigger adverse effects. mRNA vaccines also require delivery systems to achieve antigen expression in antigen-presenting cells (APCs) within lymphoid organs. Some vaccines directly target APCs in the lymphoid organs, while others rely on APCs migration to the draining lymph nodes after taking up mRNA vaccines. This review explores the current mechanistic understanding of these processes and the ongoing efforts to improve vaccine safety and efficacy based on this understanding.

## Introduction

mRNA provides a potent tool for vaccines [1]. In the case of infectious diseases, the vaccine can target various pathogens simply by altering the mRNA sequence once the mRNA delivery platform is established. This feature allowed the rapid emergency approval of two mRNA vaccines, BNT162b2 and mRNA-1273, within one year after the coronavirus disease 19 (COVID-19) outbreak caused by severe acute respiratory syndrome coronavirus 2 (SARS-CoV-2). These vaccines have demonstrated high efficacy among various vaccine modalities, playing a crucial role in controlling the pandemic [2–4]. The efficient induction of neutralizing antibodies by mRNA vaccines contributes to their high efficacy in preventing SARS-CoV-2 infection. Furthermore, mRNA vaccines evoke cellular immune responses, which are more persistent and resistant to viral mutation than humoral immune responses [5,6]. Cellular immunity may play a critical role in preventing severe diseases and deaths in COVID-19. The broad immunogenicity of mRNA vaccines, including cytotoxic T lymphocyte (CTL) induction, also makes them an attractive candidate for cancer vaccines [7]. mRNA cancer vaccines have shown promising outcomes in clinical trials by targeting shared tumour-associated antigens [8] and neoantigens derived from gene mutations in each patient [9]. The preparation of neoantigen vaccines requires rapid adaptation after identifying gene mutations in cancer and predicting suitable epitopes. In this regard, mRNA vaccines allow rapid and facile preparation corresponding to mutated proteins in each patient simply by changing the mRNA sequence. From a safety viewpoint, mRNA is rapidly degraded within a week after injection [10,11], posing a minimal risk of persistence in the body. A recent study addressed this issue in human samples, showing that mRNA became undetectable by quantitative PCR (qPCR) within a month after vaccination [12]. Scalability for billions of doses per year is another critical attribute, especially in a pandemic [13].

mRNA vaccines against infectious diseases and cancers were documented as early as the middle of the 1990s [14,15]. However, they required many years of research and technological breakthroughs before achieving current clinical success, especially in the fields of mRNA synthesis and delivery systems. In the former, pseudouridine modification of mRNA alleviated innate immune responses against mRNA and improved protein translation efficiency [16]. Other critical technologies include adding a 5’ cap and poly A tail, sequence optimization of coding and untranslated regions, and removing impurities from *in vitro* transcribed mRNA, as extensively reviewed elsewhere [17,18]. These mRNA synthesis technologies improve mRNA vaccine safety by avoiding undesirable inflammatory responses and enhance vaccine efficacy by increasing antigen production efficiency. mRNA vaccines also require delivery systems to protect mRNA from enzymatic degradation, deliver mRNA into APCs and lymphoid tissues, and facilitate intracellular mRNA trafficking to the translation site in the cytoplasm. In addition, recent studies have unveiled the role of delivery nanoparticles as an immunostimulatory adjuvant [19,20]. Notably, before the middle of the 2010s, most mRNA vaccine clinical trials employed APC transplantations after *ex vivo* mRNA delivery [21]. However, the *ex vivo* strategy requires the arduous procedure of cell culturing in clinical grades before transplantation, limiting mRNA applications to cancer immunotherapy. Recent technological advances enable effective *in vivo* mRNA delivery, expanding mRNA application to infectious disease vaccines and avoiding laborious *ex vivo* procedures in cancer vaccines. Among the various design attributes of mRNA vaccines described above, this review focuses on two issues: vaccine adjuvanticity and delivery systems targeting APCs and lymphoid organs, which are particularly important in vaccine development. Meanwhile, we redirect readers to other reviews for a general description of mRNA delivery systems [22,23], topics specific to COVID-19 vaccines [24,25], and the clinical landscape of mRNA vaccines [26].

## Adjuvanticity

### Overview of adjuvanticity

Immunostimulatory adjuvants enhance the efficiency and durability of vaccination outcomes by eliciting innate immune responses, connecting antigen expression to adaptive immunity induction [27]. The role of adjuvants has grown in importance in modern vaccines derived from recombinant proteins or nucleic acids encoding proteins. Notably, these vaccines lack many of the pathogen-associated molecular patterns (PAMPs) presented in conventional live-attenuated and inactivated vaccines, which serve as adjuvants. In the two approved mRNA COVID-19 vaccines, adjuvant functions are integrated into ionizable lipid-based lipid nanoparticles (iLNPs) formulations as built-in adjuvants rather than being supplemented as additional adjuvants [28]. Indeed, strong innate immune responses to mRNA iLNPs in mice [20] and humans [29] play crucial roles in vaccine functions [19]. Both the lipid components of iLNPs and mRNA molecules contribute to the adjuvanticity in mRNA vaccines [30]. The built-in adjuvant approach allows for a simple all-in-one formulation of mRNA vaccines [31], facilitating smooth clinical translation. Moreover, the all-in-one formulation enables the co-delivery of antigen mRNA and adjuvants into the same APCs, effectively activating APCs expressing antigen proteins from the delivered mRNA. In this regard, extensive research on recombinant protein vaccines has demonstrated the benefits of co-delivering antigens and adjuvants [32,33]. In a pioneering study, poly(D,L-lactide-co-glycolide) (PLGA) microspheres co-encapsulating CpG DNA and ovalbumin (OVA) outperformed a mixture of microspheres separately encapsulating these two components in inducing antigen-specific cytotoxic T lymphocyte (CTL) activity in mice [32]. Despite the critical roles of adjuvants in vaccination, adjuvanticity should be precisely controlled to activate the proper signalling pathway at an optimal intensity, as excessive and unintended adjuvanticity poses safety concerns and negatively impacts the vaccination effect. This section provides viewpoints on adjuvanticity in mechanistic understanding and the development of mRNA vaccines.

### Innate immune responses to current mRNA vaccines

**Figure 1.**
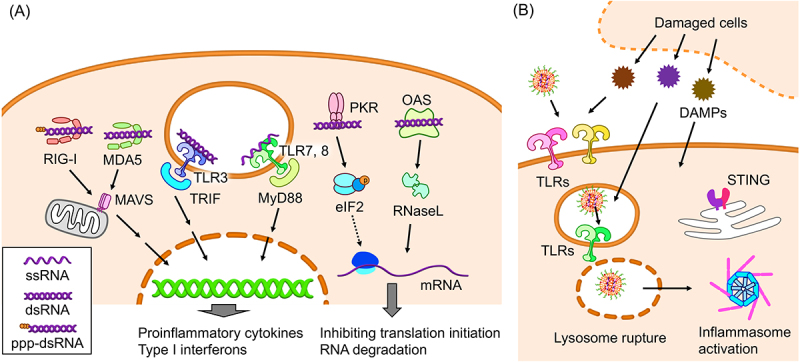
Innate immunity induction by mRNA vaccines. (a) Receptors of exogenous RNA and their signalling pathways. ppp-dsRNA: double stranded RNA possessing a triphosphate group at the 5’ end. (b) Potential mechanisms of innate immune responses to lipid components of iLNPs.

mRNA triggers innate immune responses via several innate immune receptors, including Toll-like receptors (TLRs) in the endosome and retinoic acid-inducible gene I (RIG-I) and melanoma differentiation-associated protein 5 (MDA5) in the cytosol (Figure 1(a)). Preferred ligands for each receptor are >45 base-pair (bp) double-stranded RNA (dsRNA) for TLR3 [34], >20 nucleotide (nt) single-stranded RNA (ssRNA) for TLR7, 8 [35], several-thousand bp dsRNA for MDA-5 [36], and >20 nt dsRNA with a triphosphate group at a blunt 5’ end for RIG-I [37], respectively. Major adaptor molecules for these receptors are Toll/interleukin-1 (IL-1) receptor domain-containing adaptor inducing interferon-β (IFN-β) (TRIF) for TLR3, myeloid differentiation primary response 88 (MyD88) for TLR7, 8, and mitochondrial antiviral signaling protein (MAVS) for MDA-5 and RIG-I [38]. These molecules eventually trigger the production of type I IFN and other pro-inflammatory cytokines. In addition, the 5’ cap structure of mRNA is critical for discriminating self and non-self mRNA. The 5’ cap structure is classified based on the 2’O-methylation status in the ribose of the first two nucleotides. The 2’O-methylation exists in none of the first two nucleotides in Cap0, only in the first nucleotides in Cap1, and both in the first two nucleotides in Cap2. In higher eukaryotes, mRNA has a Cap1 or Cap2 structure, leaving Cap0 mRNA recognized as non-self mainly by RIG-I [39]. A recent study revealed that the Cap2 structure further reduced the mRNA affinity to RIG-I compared to the Cap1 structure [40].

Impurities in *in vitro* transcribed mRNA, including uncapped and long dsRNA, induce strong innate immune responses. Uncapped RNA prepared by *in vitro* transcription has a triphosphate group at the 5’ terminus, which stimulates RIG-I. Although recent technologies, including CleanCap, have improved capping efficiency [41,42], a small amount of uncapped RNA produced during CleanCap mRNA preparation still significantly activates innate immune responses. Indeed, Antarctic phosphatase treatment of CleanCap mRNA solution, which removes a 5’ triphosphate group from uncapped mRNA impurity, drastically reduced cytokine responses in cultured macrophages [43]. This result highlights significant immunostimulation by uncapped mRNA contaminants in CleanCap mRNA. Long dsRNA, a strong inducer of innate immunity [44], is prepared from RNA-templated RNA transcription during *in vitro* transcription. In this process, an intended mRNA strand prepared from a DNA template functions as a template to synthesize a complementary unintended RNA strand [45,46], resulting in the formation of long dsRNA. As discussed later, innate immune responses to these impurities may drastically impact the outcome of mRNA vaccination [47], requiring careful discussion about vaccination effects in light of the immunostimulation by the impurities.

Although mRNA vaccines require innate immune responses as an adjuvant, excessive responses pose safety concerns and negatively impact the vaccination effect, as described later. Furthermore, cellular responses to exogenous mRNA negatively affect protein expression efficiency from mRNA by inducing global mRNA degradation and impairing translation initiation (Figure 1(a)). mRNA recognition by 2’−5’-oligoadenylate synthetases (OAS) and following RNase L activation cause massive intracellular ssRNA degradation [48]. In addition, exogenous RNA impairs the initiation process of protein translation by activating RNA-dependent protein kinase (PKR) and phosphorylating translation initiation factor 2-α (eIF-2α) [49]. Therefore, the development of mRNA vaccines requires controlling the cell sensing of *in vitro* transcribed mRNA. For this purpose, numerous mRNA vaccines use nucleoside modification with pseudouridine (Ψ) or N1-methyl-pseudouridine (1 mΨ), which alleviates innate immune responses to mRNA [16,38]. In addition, eliminating long dsRNA using high-performance liquid chromatography (HPLC) and removing 5’ triphosphate from uncapped mRNA by Antarctic phosphatase also reduce immunostimulation by mRNA impurities [38,43]. More recent technologies of mRNA purification include cap analogs possessing a photocleavable hydrophobic tag for purifying capped mRNA from uncapped mRNA [50] and engineered RNA polymerases for minimizing dsRNA production [51]. These approaches could improve the safety and efficacy of mRNA vaccines by increasing antigen protein translation and mitigating pro-inflammatory responses.

In iLNP formulations, a widely used platform for current mRNA vaccines, their lipid components are primary inducers of innate immune responses. Indeed, empty iLNPs without mRNA encapsulation induced strong pro-inflammatory responses after local injection into mice [19,52]. Notably, the pro-inflammatory responses became almost absent after the removal of the ionizable lipid component, showing the critical role of ionizable lipids in immunostimulation. Ionizable lipids have a neutral charge at extracellular pH and become cationic at endo/lysosomal pH. Therefore, ionizable lipids had been considered less inflammatory than conventional cationic lipids without pH responsiveness because using ionizable lipids instead of cationic lipids may reduce LNP interaction with the cell membrane and subsequent membrane damage [22]. Conversely, substituting ionizable lipids in iLNP with cationic lipids reduced the innate immune responses against the nanoparticles [19]. Several reports provided mechanistic insights into such innate immune activation by iLNP (Figure 1(b)). As described later, several ionizable lipids specifically activate innate immune receptors, including TLRs and the intracellular stimulator of interferon genes (STING) [53–55]. In addition, studies of mRNA iLNPs and other nanoparticles showed that lysosomal rupture, an essential intracellular process of mRNA delivery, elicits innate immune responses via inflammasome activation [56,57]. Damage-associated molecular patterns (DAMPs) released from damaged cells at the injection site, including DNA, RNA, and proteins, may also stimulate innate immune receptors after iLNP delivery. Indeed, a recent animal study revealed a drastic increase in the serum levels of DAMPs, including double-stranded DNA, high-mobility group box (HMGB) 1, and uric acid, after intramuscular (i.m.) injection of BNT162b2, an approved COVID-19 mRNA vaccine [20]. In humans, the approved COVID-19 mRNA vaccines induced strong local reactions [3,4], presumably causing the release of DAMPs.

### Bridging adjuvanticity to vaccination outcomes

This section addresses how innate immune responses influence vaccination outcomes, focusing on iLNPs. A recent comprehensive study investigated the involvement of various innate immune receptors in inducing humoral and cellular immunity after vaccination with BNT162b2, an approved COVID-19 mRNA vaccine, by preparing knockout mice for various receptors [20]. The target receptors include TLR3, TLR7, RIG-I, and MDA-5 in the RNA sensing pathway, NLR family PYD domain-containing protein (NLRP) 3 and apoptosis-associated speck-like protein containing a C-terminal caspase recruitment domain (ASC) in the inflammasome, and TLR2, TLR4, TLR5, cyclic GMP-AMP synthase (cGAS), and STING, some of which are potential target molecules of ionizable lipid-mediated immunostimulation. Among these genes, only the knockout of *MDA-5* drastically reduced the frequency of spike antigen-specific CD8^+^ T cells after vaccination. More precisely, *MDA-5* knockout diminished the secretion of IFN-α, a downstream molecule of MDA-5 signalling, and prevented the activation of dendritic cells. Furthermore, the knockout of the *IFN-alpha/beta receptor (IFNAR) 1* also impaired spike-specific CD8^+^ T cell induction. These results reveal a critical role of MDA-5 and IFN-α in the induction of CD8^+^ T cell responses. MDA-5 May recognize dsRNA in mRNA vaccine formulations or dsRNA released from damaged cells as DAMPs.

However, the pathways for humoral immunity induction are more complicated. Knockout of any innate immune receptor listed above did not reduce the efficiency of spike-specific antibody induction. In addition, only a minimal reduction of spike-specific antibodies was detected after *IFNAR1* knockout. This result is consistent with that from a human study [58]. The human study evaluated the efficacy of COVID-19 mRNA vaccines in patients with congenital and acquired deficiency of type I interferon signalling caused by inherent deficiency of *TLR7*, *IFNAR1*, or *interferon regulatory transcription factor (IRF) 7*, or autoantibody to type I IFNs. mRNA vaccines efficiently induced neutralizing antibodies and memory B cells in these patients at levels comparable with those in healthy controls. Meanwhile, several animal studies provided insights into the pathways critical for humoral immunity induction. The knockout of *MyD88*, a gene encoding an adaptor protein of many TLRs, drastically suppressed the induction of antigen-specific follicular helper T (Tfh) and germinal centre (GC) B cells after vaccination using mRNA iLNP [19]. In addition, the knockout of *interleukin* (*IL)-6*, a gene encoding a critical cytokine in Tfh cell differentiation, also eliminated the vaccination effect. In contrast, the vaccination effects were maintained after the knockout of *MAVS*, a gene encoding an adaptor protein of RIG-I and MDA-5. Another study of self-amplifying RNA (saRNA) also demonstrated the role of IL-6 in mouse vaccination [59], showing that *IL-6* knockout impaired the antibody responses and induction of Tfh cells and GC B cells. Meanwhile, the protective effect of saRNA vaccines against lethal influenza virus challenge was preserved after *IL-6* knockout. These studies indicate redundancy in the innate immunity signalling pathway for inducing humoral immune responses, while TLRs and IL-6 play predominant roles.

In human vaccination, the second dose of COVID-19 mRNA vaccines drastically enhances humoral and cellular immunity and concurrently induces more severe systemic reactogenicity than the first dose [3,4]. This observation implies that pro-inflammatory molecules responsible for the reactogenicity after the second dose might also contribute to boosting adaptive immunity. A previous study evaluated the plasma profile of 67 cytokines in human BNT162b2 vaccinees. Among them, IFN-γ showed a distinctive profile with a drastic increase, especially after the second dose [29]. Mouse models also reproduced a drastic increase in IFN-γ levels after the second dose of BNT162b2, accompanied by the activation of macrophages, monocytes, and dendritic cells [20]. Further analyses revealed a substantial contribution of CD4^+^ and CD8^+^ T cells in producing IFN-γ after the second dose. Intriguingly, administration of an IFN-γ receptor neutralizing antibody dampened the activation of macrophages, monocytes, and dendritic cells, demonstrating an adjuvant functionality of IFN-γ after the second dose. However, an IFN-γ receptor neutralizing antibody minimally impacted the efficiency of antigen-specific antibody production and CD4^+^ and CD8^+^ T cell induction. Thus, further studies are required to decipher the adjuvanticity mechanisms of COVID-19 mRNA vaccines.

### Adjuvanticity of lipids and mRNA

Another key question in iLNP adjuvanticity is which of the lipid and mRNA components predominantly contributes to immunostimulation and what signalling pathways each component activates. A comprehensive mechanistic study addressed this issue by comparing empty iLNP with iLNP encapsulating modified mRNA [19]. Interestingly, both formulations produced similar levels of cytokines and chemokines in the draining lymph nodes 4 and 24 h after intradermal (i.d.) injection into mice, indicating the predominant role of lipid components in innate immune responses against iLNPs. As a result, both iLNPs functioned as adjuvants and improved the efficacy of recombinant protein vaccines to produce antigen-specific antibodies more efficiently than AddaVax, a squalene adjuvant mimicking a clinically approved MF59 adjuvant. Interestingly, the adjuvant effect of empty iLNP was mostly maintained after the knockout of *MyD88* and *MAVS* yet decreased after *IL-6* knockout. Therefore, empty iLNP may exert its adjuvanticity through IL-6 secretion. However, detailed analyses in the same study revealed the contribution of mRNA to innate immune responses to mRNA iLNPs, even when mRNA was chemically modified to minimize immunostimulation. When empty iLNP and iLNP loading mRNA were compared for their adjuvanticity to improve the efficacy of recombinant protein vaccines, the knockout of *MyD88*, a gene encoding an adaptor protein of many TLRs, reduced humoral immunity induction in iLNP loading mRNA but not in empty iLNP. This result indicates that mRNA, rather than lipid components, activates MyD88. In another study, after i.m. and i.d. injection into non-human primates (NHPs), only iLNP encapsulating modified mRNA, not empty iLNP, induced type I IFN expression in dendritic cells in the draining lymph nodes and maturation of the dendritic cells [60], revealing an essential role of mRNA in iLNP adjuvanticity.

The adjuvanticity of mRNA becomes more evident with the use of unmodified mRNA and less immunostimulating mRNA delivery carriers. In this regard, the anionic lipoplex used in cancer vaccine clinical trials is less immunostimulatory than iLNPs [61]. Interestingly, the anionic lipoplex provides robust CTL activity when loading unmodified mRNA [8,9,62] but conversely induces antigen-specific immune tolerance when loading 1 mΨ mRNA [63]. These studies highlight the adjuvanticity of unmodified mRNA to convert immune tolerance vaccines into anti-cancer vaccines. In the anionic lipoplex loading unmodified mRNA, the knockout of *TLR7* and *IFNAR1*, but not that of *TLR3*, *4*, and *9*, reduces the activation of dendritic cells, NK cells, T cells, and B cells after vaccination [62]. This result indicates the contribution of mRNA recognition by TLR7 and subsequent activation of type I IFN in the adjuvanticity of unmodified mRNA.

### Designing delivery systems for improving adjuvanticity

The mechanistic understanding reviewed so far implies the benefit of improving adjuvanticity in mRNA vaccines, prompting vigorous research in this field (Table 1). Indeed, in some settings, adjuvanticity, rather than antigen expression efficiency, may determine vaccination efficacy, wherein a vaccine providing enhanced immunostimulation and lower antigen expression outperformed that providing less efficient immunostimulation and enhanced antigen expression [76,90–92]. In designing mRNA vaccine adjuvants, all-in-one vaccine formulations containing antigen mRNA and adjuvants are preferred, allowing co-delivery of antigens and adjuvants. As described above, research on recombinant protein vaccines has shown the benefit of co-delivering antigen and adjuvant for robust vaccination [32,33]. In addition, such simple vaccine formulations are favourable in clinical translation.

**Table 1.** Strategies to improve or modulate adjuvanticity in mRNA vaccines.

Category	Technologies	Target pathways	Applications	Ref.
Co-administration of adjuvants	GM-CSF	-	Renal cell cancer	[64]
	Fms-like tyrosine kinase 3 (FLT3) ligand	FLT3 (DC activation)	Melanoma	[65]
	R848	TLR7	Influenza virus	[66]
Lipid nanoparticles encapsulating hydrophilic adjuvants	iLNP encapsulating manganese	STING	SARS-CoV-2	[67]
	PLGA-core/lipid shell nanoparticles containing gardiquimod	TLR7	Cancer (model antigen)	[68]
Lipid nanoparticles containing hydrophobic adjuvants	Lipid conjugated with R848	TLR7, 8	Cancer (model antigen)	[69]
	iLNP containing Pam2Cys	TLR2/6	Colon cancer, SARS-CoV-2	[70]
	Lipid carriers containing α-galactosylceramide	iNKT cell activation	Cancer (model antigen)	[71]
			Malaria	[72]
			Melanoma	[73]
	Monophosphoryl lipid A	TLR4	-	[74]
			Cancer (model antigen)	[75]
Immunostimulatory ionizable/cationic lipids	Ionizable lipids with a cyclic amine head group	STING	Cancer (model antigen)	[53]
			SARS-CoV2	[76]
	Cationic lipid-like material	TLR4	Melanoma	[54]
	An adjuvant ionizable lipidoid conjugated with a TLR7, 8 adjuvant	TLR7, 8	SARS-CoV-2	[55]
	Ionizable lipids with vitamin E in the tail	-	Toxoplasma gondii	[77]
	Ionizable lipid conjugated with STING ligand	STING	SARS-CoV-2	[78]
Inorganic systems	A lipid carrier calcium phosphate core	Ca^2+^ signalling	Melanoma	[79]
Polymeric systems	Protamine/mRNA polyplex	TLR7	Cancer (model antigen)	[84]
	Mannan-coated polymeric nanoparticle	-	Cancer (model antigen)	[81]
	Cationic chitosan	STING, inflammasome	Cancer (model antigen)	[82]
	Polycation with C18 side chains	-	Cancer (model antigen)	[83]
mRNA encoding pro-inflammatory genes	CD40 ligand, constitutively active TLR4, CD70	CD40, TLR4, CD70	Melanoma, myeloid leukaemia, mastocytoma	[84]
	Constitutively active STING mRNA	STING	Human papillomavirus positive cancer	[85]
	mRNA encoding C3d	Complement	SARS-CoV2	[76]
Inhibiting immune responses to saRNA	MERS-CoV-2 ORF4	Inhibiting the activation of MDA-5 and RIG-I	Rabies virus	[86]
	Vaccinia virus immune evasion proteins	Inhibiting the activity of PKR and IFN-β	-	[87]
dsRNA hybridization	Poly U hybridization to poly A of mRNA	RIG-I, TLR3	-	[88]
	Comb-structured mRNA	RIG-I	Melanoma	[89]

**Figure 2.**
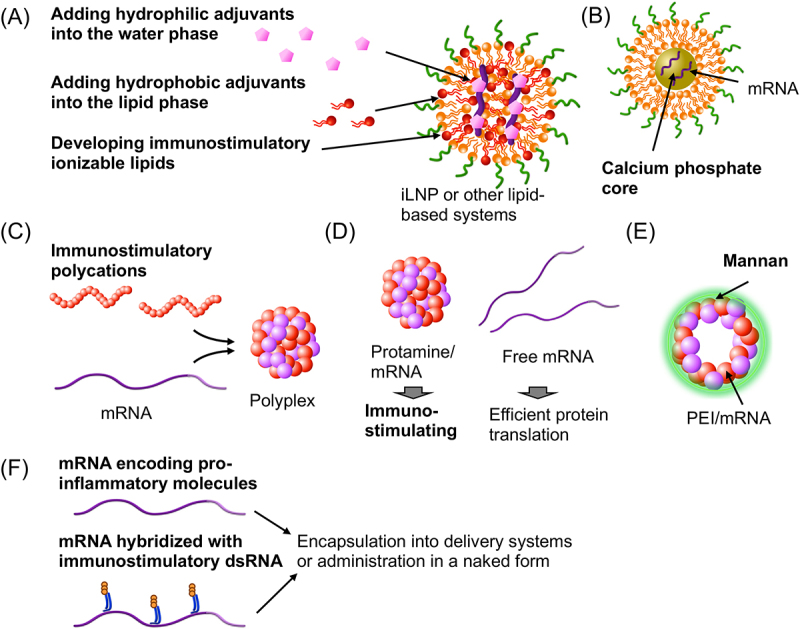
Strategies to improve adjuvanticity of mRNA vaccines. (a) iLNPs or other lipid systems. (b) A lipid carrier containing a calcium phosphate core. (c) Polyplexes. (d) Protamine/mRNA polyplexes. (e) A hollow-core sugar capsule coated with mannan. (F) Designing mRNA for improving adjuvanticity. Strategies and components to enhance adjuvanticity are highlighted in bold.

One strategy is the co-encapsulation of adjuvants and mRNA into the same nanoparticles. Lipid-based systems have two options: encapsulating hydrophilic adjuvants in the water phase or hydrophobic adjuvants in the lipid phase (Figure 2(a)). An example of the former approach is iLNP encapsulating manganese, an activator of the STING pathway, which shows promising outcomes in vaccination against SARS-CoV-2 [67]. In the latter approach, R848, a small-molecule TLR7/8 agonist, was conjugated with palmitic acid for incorporation into cationic mRNA lipoplex [69]. The lipoplex exhibited robust CTL induction and anti-cancer effects. In another example, Pam2Cys, a TLR2/6 adjuvant consisting of cysteine, thioglycerol, and two palmitic acid chains, was incorporated into iLNP, thereby enhancing cellular and humoral immune responses [70]. Other systems utilized α-galactosylceramide, which activates invariant natural killer T (NKT) cells after presentation onto APCs, demonstrating their utility in vaccines against cancer [71] and malaria [72]. The malaria vaccine targeted infected hepatocytes.

Besides using additional adjuvants, incorporating adjuvanticity into ionizable or cationic lipids is an option in lipid-based vaccines (Figure 2(a)). Without additional adjuvants, this approach simplifies the vaccine formulation by minimizing components, potentially benefitting clinical translation. The adjuvant lipids have dual functionalities of activating innate immune responses and facilitating mRNA delivery. For instance, ionizable lipids with a cyclic amine head group induced innate immune responses and antigen-specific CTL activity against cancer more efficiently than those with a linear amine head group [53]. Interestingly, the knockout of STING eliminated the innate and adaptive immune responses induced by the cyclic amine iLNP, suggesting that the cyclic amine in the ionizable lipid activates STING. A more recent study introduced a biodegradable ester linker between a cyclic amine and alkyl chains to facilitate the clearance of the adjuvant cyclic amine for safety [76]. Another study developed a cationic lipid-like material that activates TLR4 [54]. The resulting lipid-based vaccine improved cellular immunity in wild-type mice but not in *TLR4* knockout mice, demonstrating the involvement of TLR4 signalling in the vaccine. Conjugating existing adjuvants or their components to ionizable lipids is a more straightforward approach. A recent study designed an adjuvant ionizable lipidoid conjugated with a TLR7/8 adjuvant, which improved the induction efficiency of cellular and humoral immunity [55]. Another study introduced vitamin E, a component of a clinically approved AS03 adjuvant, into the hydrophobic tail of ionizable lipids [77]. The ionizable lipid with vitamin E exhibited enhanced adjuvanticity to induce CTL responses more efficiently than the control ionizable lipids possessing myristic acid or oleic acid tail.

Using inorganic systems in lipid-based formulations offers another example of dual-functional systems with delivery and adjuvant functionalities. A previous study designed nanoparticles comprised of mRNA-loaded calcium phosphate core coated with a lipid layer (Figure 2(b)). The calcium phosphate core stabilizes the nanoparticles in the extracellular environment but releases Ca^2+^ inside the cells [79]. The resulting increase in intracellular Ca^2+^ concentration triggers signalling for dendritic cell maturation. Moreover, the calcium phosphate core can co-encapsulate *PD-L1* siRNA with antigen mRNA for immune checkpoint blockade, further potentiating the cancer vaccination effect.

Polymer-based systems, a large class of mRNA delivery systems [23,93], have attracted less attention as mRNA vaccine formulations than lipid-based systems. A previous study compared the vaccination efficacy of saRNA between a polyplex and iLNP [91]. Interestingly, iLNP loading saRNA far outperformed the polyplex loading saRNA in cellular and humoral immunity induction after i.m. delivery, even though the polyplex provided more efficient antigen expression than iLNP. Consistently, iLNP is more potent than the polyplex in inducing the expression of IL-6, a key molecule of mRNA vaccine adjuvanticity [19]. Therefore, the authors explain the low vaccination efficiency of the polyplex by its low adjuvanticity, although other factors, including delivery efficiency to the lymphoid tissues, may also impact vaccination outcomes.

Meanwhile, some polyplex systems provide promising results in vaccinations (Figure 2(c)). Protamine/mRNA polyplexes used in several clinical trials utilize the adjuvanticity of unmodified mRNA to stimulate TLR7 (Figure 2(d)). In this system, mRNA complexation with protamine increased innate immune responses but decreased the antigen expression efficiency compared to naked mRNA after i.d. injection into mice [80]. Thus, the mixing ratio of mRNA and protamine was controlled to include free mRNA and protamine/mRNA polyplexes in the injected solution to attain antigen expression and adjuvanticity simultaneously. In a mechanistic study, *TLR7* knockout almost completely suppressed IL-12 secretion after the polyplex administration. Furthermore, the polyplex reduced its CTL induction potential by the knockout of *TLR7* but not by that of *TLR9*, highlighting the role of TLR7 adjuvanticity in this vaccine formulation [94]. In another example, a silica nanoparticle was coated with poly(ethyleneimine) (PEI) followed by cross-linking of PEI, mRNA loading, and mannan coating [81]. Then, the silica core was removed, providing hollow-core sugar capsules (Figure 2(e)). The mannan functions to target dendritic cells and activate dendritic cells as an adjuvant. As a result, this system provided robust CD4^+^ and CD8^+^ immunity for cancer vaccines. Recent studies also showed the potential of adjuvant polycations in mRNA vaccines (Figure 2(c)). For instance, a 200-kDa cationic chitosan used for mRNA complexation also served as an adjuvant to potentiate antigen-specific cellular and humoral immunity by activating STING and the inflammasome [82]. In another study, a polycation with C18 side chains exhibited efficient cancer vaccination effects due to its adjuvanticity to activate dendritic cells and efficient lymph node migration [83]. A study of subunit vaccines also unveiled polycation adjuvanticity. In this study, PEI exerted its adjuvanticity after intranasal delivery by activating IFN regulatory factor-3 signalling by releasing DNA from host cells as DAMPs [95]. These studies showcase the potential of polymer-based systems in vaccination, which has not been fully explored. As mentioned earlier, iLNP also generates DAMPs [20], which may serve as an adjuvant. Meanwhile, an excess of DAMPs could trigger autoimmune disorders. For instance, overactivation of the cGAS-STING pathway by self-DNA can potentially cause Aicardi – Goutières syndrome and ataxia telangiectasia [96]. Vaccine development needs to consider these risks.

### Designing mRNA for improving adjuvanticity

Modulating mRNA adjuvanticity provides another option to improve the vaccination effect. This mRNA adjuvant approach includes adding mRNA encoding immunostimulatory proteins and leveraging the intrinsic immunostimulatory properties of RNA molecules (Figure 2(f)). These strategies allow the co-delivery of antigen mRNA and adjuvant RNA using a single nanoparticle formulation. In addition, adjuvant RNA undergoes enzymatic degradation within several days after injection, avoiding prolonged immunostimulation [11,12]. Regarding mRNA encoding immunostimulatory proteins, a pioneering study mixed antigen mRNA with three types of immunostimulatory mRNA encoding CD40 ligand, a constitutively active form of TLR4, and CD70. This design drastically improved antigen-specific CD4^+^ and CD8^+^ T cell induction and anti-cancer effect compared to the injection of antigen mRNA alone in a naked form after intranodal injection [84]. Recent studies demonstrated the benefit of immunostimulatory mRNA in iLNP formulations. For instance, a previous study identified constitutively active STING mRNA as the most effective mRNA to induce CTL activity in mice by screening various mRNA species encoding proteins involved in innate immune signalling [85]. Another study employed mRNA encoding C3d, a mammalian complement component, which activates follicular dendritic cells and B cells [76]. C3d mRNA encapsulation into iLNP improved antigen-specific cellular and humoral immunity.

The intrinsic immunostimulatory properties of RNA molecules, especially those of dsRNA, can be utilized as adjuvants. In this regard, the immunostimulatory properties of dsRNA can positively or negatively impact the vaccination efficacy of saRNA vaccines. saRNA possesses the replication machinery of single-stranded RNA viruses for self-amplification inside host cells, providing prolonged expression of antigen proteins [1,87]. Furthermore, dsRNA intermediates during intracellular proliferation may also enhance cellular dsRNA receptor activation to induce innate immune responses. These features enable effective vaccination in animals and humans using low doses of saRNA [97,98]. However, the persistence of dsRNA intermediates may cause prolonged immunostimulation and PKR activation, potentially posing negative impacts on vaccination outcomes in some settings. Indeed, the knockout of *IFNAR1* or *MAVS* improved the efficiency of humoral or cellular immunity induction after saRNA vaccination [99,100]. In addition, saRNA improved its vaccination efficiency by co-expression of MERS-CoV-2 ORF4a, which inhibits the activation of MDA-5 and RIG-I by binding to dsRNA [86] or vaccinia virus immune evasion proteins blocking the activity of PKR and IFN-β [87].

**Figure 3.**
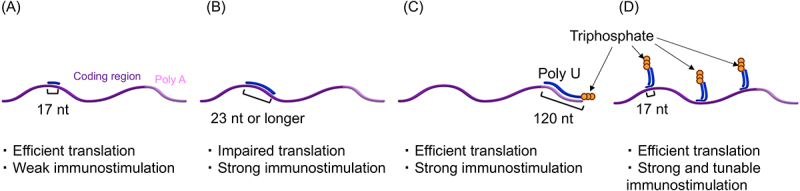
mRNA hybridized with dsRNA adjuvants. Translation efficiency and immunostimulatory properties after RNA hybridization are depicted. (a-c) Complementary RNA was hybridized to the coding region of mRNA (a, b) and poly a (c). (d) Comb-structured mRNA hybridized with dsRNA designed to target RIG-I in the coding region of mRNA.

In contrast, transient stimulation of the innate immune response using dsRNA benefits mRNA vaccines to improve CTL activity. In this strategy, the dsRNA structure is prepared in non-replicating mRNA by hybridizing complementary RNA to mRNA (Figure 3). Previous studies evaluated the influence of complementary RNA hybridization to mRNA in terms of protein translation efficiency and innate immune responses. When complementary RNA targets the protein-coding region, translation efficiency remained stable after the hybridization of 17 nt complementary RNA (Figure 3(a)) but decreased after the hybridization of a 23 nt or longer RNA (Figure 3(b)) [101]. Short RNA hybridized to mRNA was detached from mRNA specifically in the intracellular environment, presumably via the endogenous helicase activity of the protein translation machinery during the translational process [102]. This process may allow efficient protein expression from mRNA hybridized with 17 nt complementary RNA strands. Regarding adjuvanticity, the hybridization of a 23 nt or longer complementary RNA, but not a 17 nt complementary RNA, increases the immunostimulatory properties of mRNA. Meanwhile, the influence of RNA hybridization is different when the non-coding region of mRNA is targeted. In a previous study, 120 nt poly U RNA prepared by *in vitro* transcription was hybridized to 120 nt poly A of mRNA [88]. Intriguingly, the poly U hybridization drastically enhanced mRNA capability of inducing innate immune responses with minimal influence on translational efficiency (Figure 3(c)), providing an excellent formulation of mRNA with adjuvanticity. Mechanistic analyses revealed the predominant role of RIG-I in innate immunity activation. Indeed, the 120 nt poly U RNA was *in vitro* transcribed without a 5’cap analog, possessing a triphosphate group at the 5’ end, a ligand of RIG-I. In mouse vaccination, this formulation enhanced vaccination effects after intranodal delivery of model antigen mRNA in a naked form. However, the intensity of immunostimulation is not controllable in this system. Thus, a later study re-designed this system to enable fine-tuning of immunostimulation intensity [89]. For this purpose, comb-structured mRNA was prepared by hybridizing mRNA with a controlled number of dsRNA strands via a 17 nt complementary RNA (Figure 3(d)). The dsRNA teeth were precisely designed to induce RIG-I stimulation. More importantly, by changing the number of immunostimulatory teeth, this strategy enables fine-tuning of adjuvanticity to maximize vaccine efficacy with minimal adverse effects. Ultimately, this system improved the CTL activity of three different mRNA formulations: an anionic lipoplex, iLNP, and a PEGylated polyplex.

### Jekyll and Hyde nature of adjuvanticity

**Figure 4.**
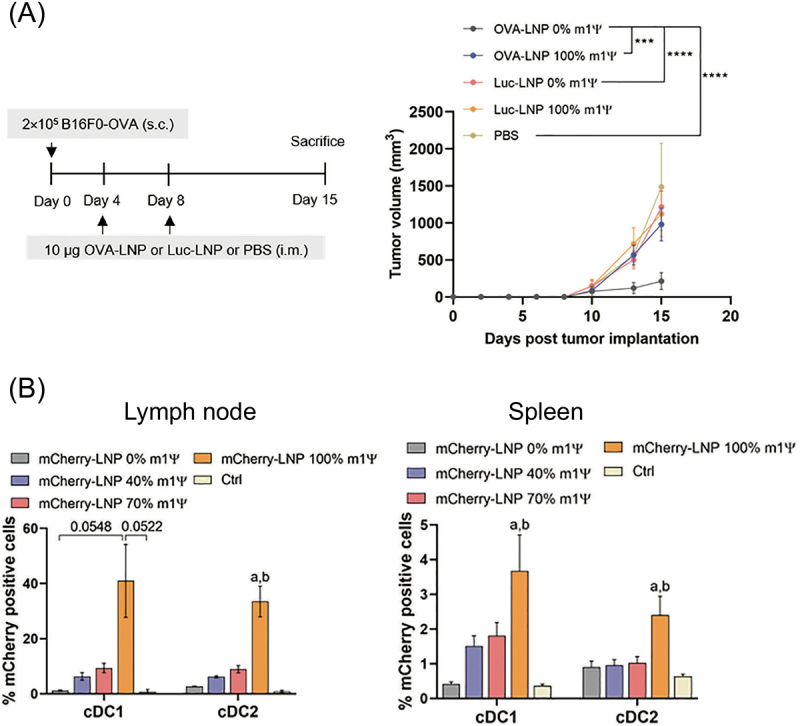
Effects of 1 mΨ modification on cancer vaccination. (a) iLNP loading ovalbumin (OVA) mRNA was intramuscularly injected into mice bearing subcutaneous B16F0 tumors expressing OVA. Tumor sizes are evaluated. (b) mCherry mRNA with 0, 40, 70, and 100% 1 mΨ modification was intramuscularly injected using iLNPs. The efficiency of mCherry expression in conventional type 1 and 2 dendritic cells (cDC1 and cDC2) in the lymph nodes and spleen was quantified. Reproduced from [92] under a creative commons attribution license (CC BY). Copyright © 2022 sittplangkoon, Alameh, Weissman, Lin, Tam, Prompetchara and Palaga.

The description above focuses on the positive aspects of innate immune responses in mRNA vaccination. However, numerous studies have revealed more complicated facets of adjuvanticity. For example, mRNA modification provides complicated outcomes in vaccination. mRNA modifications, such as Ψ and 1 mΨ, mitigate innate immune responses against mRNA, *i.e*., adjuvanticity. The adjuvanticity of unmodified mRNA produces opposite effects on CTL induction and antibody production. Several iLNP studies have shown the benefit of unmodified mRNA over modified mRNA in inducing antigen-specific CD8^+^ T cell responses [92,103]. According to a recent study, iLNP encapsulating antigen mRNA without nucleoside modification induced antigen-specific CD8^+^ T cells, including those positive for granzyme B, and activated tumour-infiltrating dendritic cells more efficiently than iLNP encapsulating 1 mΨ-modified antigen mRNA [92]. As a result, unmodified mRNA outperformed modified mRNA in cancer vaccines in mouse models, showing efficient suppression of tumour volume and prolonged survival of mice (Figure 4(a)). Strikingly, in a reporter assay using mRNA encoding a fluorescent protein, unmodified mRNA provided a much lower efficiency of reporter protein expression in dendritic cells and macrophages in the lymph node and spleen than 1 mΨ-modified mRNA (Figure 4(b)). These results demonstrate the potential of unmodified mRNA in inducing CTL responses, even at the expense of antigen expression efficiency. In mechanistic analyses, unmodified mRNA activated splenic dendritic cells more efficiently than modified mRNA, and administration of an anti-IFNAR1 antibody diminished the benefit of unmodified mRNA in vaccination. Therefore, unmodified mRNA may exert its adjuvanticity via the type I IFN pathway.

**Figure 5.**
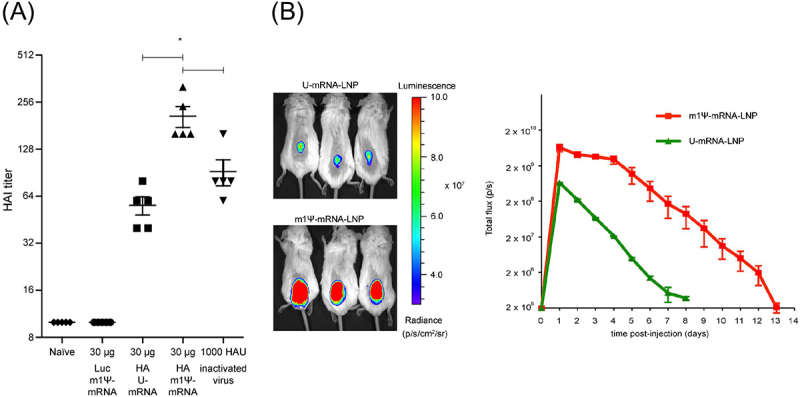
Effects of 1 mΨ modification on vaccines against infectious diseases. (a) Mice were intradermally vaccinated with unmodified or 1 mΨ-modified mRNA expressing hemagglutinin (HA) of the influenza virus using iLNPs. HA inhibition titer was evaluated. (b) Luciferase expression efficiency was evaluated after intradermal injection with iLNP loading unmodified and 1 mΨ modified mRNA. Reproduced from [104] under a creative commons attribution license (CC BY-NC-SA). Copyright © 2018 Pardi et al.

On the contrary, 1 mΨ mRNA delivered with iLNP was more effective in producing antigen-specific antibodies than unmodified mRNA, suggesting the negative impact of adjuvanticity in unmodified mRNA. A comprehensive study investigated the effect of 1 mΨ modification on the outcomes of iLNP-based influenza virus vaccines in mice [104]. After i.d. vaccination, 1 mΨ mRNA outperformed unmodified mRNA in inducing antigen-specific CD4^+^ T cells and antibodies (Figure 5(a)), accompanied by enhanced production of Tfh and GC B cells in the spleen. These results might reflect the negative influence of unmodified mRNA adjuvanticity. Meanwhile, 1 mΨ mRNA produced more efficient and prolonged reporter protein expression than unmodified mRNA (Figure 5(b)). Notably, the benefit of 1 mΨ modification on protein expression efficiency varied between organs and cell types, being highly evident, especially in splenic immune cells, including monocytes, macrophages, and dendritic cells [105]. Therefore, the capability of 1 mΨ-modified mRNA to provide enhanced antigen expression in immune cells may also contribute to its high vaccination potential. A more recent study provided mechanistic insight into the correlation between innate immune responses and antibody production efficiency by investigating the effect of 1 mΨ modification using three different iLNPs in mice and NHPs [106]. In the study, 1 mΨ modification increased the antibody production efficiency with a concomitant reduction in the systemic secretion of chemokines and cytokines. More specifically, among the three iLNPs, two iLNPs that induced intensive type I IFN secretion without 1 mΨ modification obtained a larger benefit of vaccination efficiency after 1 mΨ modification than that in the other iLNP showing minimal type I IFN responses without modification. Therefore, type I interferon responses might be a culprit that dampens the vaccination outcomes.

Immunostimulation by mRNA impurities may also have a negative impact. In a recent study of the iLNP mRNA vaccine, C57BL6 mice exhibited much weaker induction of humoral immunity than BALB/c mice [47]. In comparison between these two strains, dendritic cells from C57BL6 mice produced more than a 10-fold larger amount of type I IFN than those from BALB/c mice. After the removal of dsRNA contaminants from mRNA using HPLC, the IFN production in C57BL6 was suppressed, and antibody production in C57BL6 mice increased to a level comparable with that observed in BALB/c mice. These data also indicate the inhibitory effect of type I IFN on humoral immunity induction. In humans, deficiency in the type I IFN signalling pathway did not impair antibody production in SARS-CoV-2 mRNA vaccines, as described above [58]. Thus, antibody production in humans may not require strong adjuvanticity and type I IFN activation. From a safety viewpoint, 1 mΨ modification mitigated the systemic release of cytokines and chemokines after mRNA vaccination in NHPs using iLNP [106]. Therefore, mRNA vaccine formulation inducing minimal type I IFN responses may be practically preferable for vaccines focusing on humoral immunity. Meanwhile, it should also be noted that the necessity of chemical modification is still debatable. Indeed, iLNP loading unmodified mRNA induced effective humoral immunity in NHPs at a level comparable with that induced by the clinically approved COVID-19 vaccine loading 1 mΨ mRNA [107].

Besides nucleoside modification, type I IFN signalling on CTL induction provides another example of complicated mechanisms underlying adjuvanticity. Notably, type I IFN signalling induced opposite effects on CTL induction, depending on vaccine administration routes. *IFNAR1* knockout or IFNAR1 blocking antibody improved the CTL induction efficiency of mRNA lipoplexes after local delivery, including i.d., subcutaneous (s.c.), and intranodal delivery, showing a negative impact of type I IFN signalling on vaccination [108,109]. Importantly, IFNAR1 blockade improved the vaccination effect even without a large increase in protein expression efficiency from mRNA. Therefore, mechanisms other than antigen expression efficiency, presumably the excessive adjuvanticity of type I IFN signalling, may impair the vaccination effect. On the contrary, *IFNAR1* knockout reduces the efficiency of CTL induction after systemic injection, showing the positive impact of type I IFN adjuvanticity [110]. According to a later mechanistic study of type I IFN signalling in vaccination, both the negative effect after local delivery and the positive effect after systemic delivery were eliminated after conditional *IFNAR1* knockout in CD4^+^ T cells but not in dendritic cells [111]. Therefore, type I IFN action on CD4^+^ T cells may explain its conflicting effect on vaccination.

For CD4^+^ T cell activation, the kinetics of type I IFN signalling and antigen presentation play critical roles in the fate of CD4^+^ T cells [112]. Type I IFN signalling preceding antigen presentation to T cell receptors poses anti-proliferative and apoptotic effects on T cells. On the other hand, concomitant or delayed type I IFN signalling is proliferative and anti-apoptotic to T cells. The difference in this kinetics may explain the conflicting effect of type I IFN between systemic and local delivery. Systemic injection might deliver mRNA directly into splenic dendritic cells, allowing fast and concomitant antigen presentation relative to type I IFN exposure, thereby activating T cells. On the other hand, local injection requires time for tissue-residual APCs to migrate to lymphoid organs. This might delay antigen presentation, thereby negatively affecting T cell functioning.

**Figure 6.**
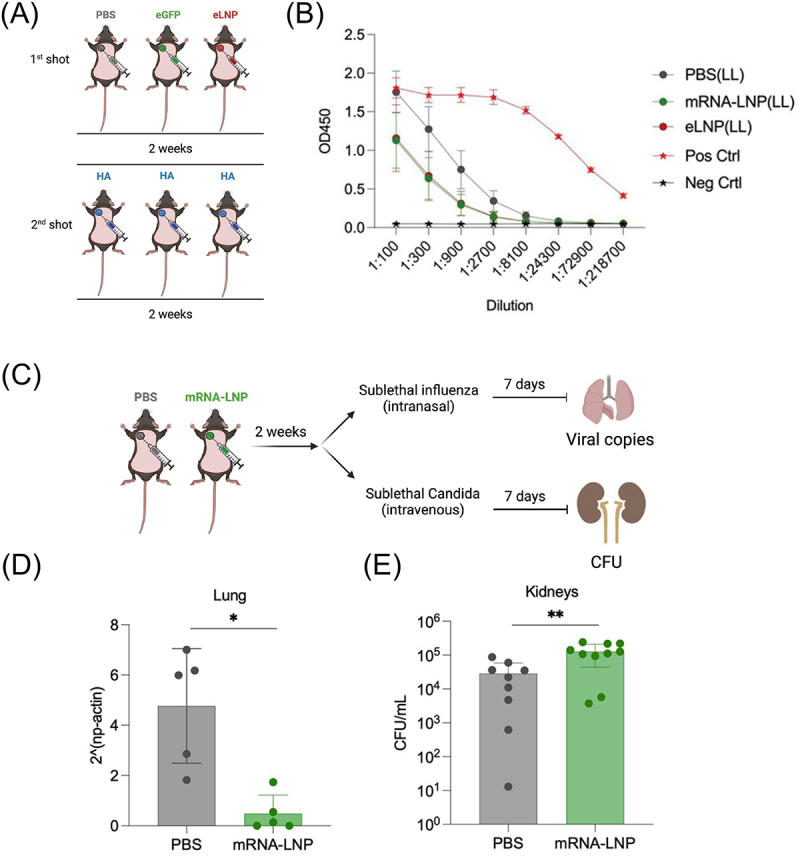
Influences of iLNP pre-injection on later innate and adaptive immune responses. (A-B) iLNP loading non-antigen mRNA (eGFP), empty iLNP (eLNP), or PBS was intradermally injected. Two weeks later, iLNP loading hemagglutinin (HA) was injected, followed by the evaluation of vaccination effects 2 weeks after the vaccination. (a) Experimental scheme. (b) HA-specific antibody production. (c-e) Two weeks after injection of iLNP loading non-antigen mRNA, mice were challenged with influenza virus or Candida albicans. (c) Experimental scheme. (d, e) viral load after the challenge with influenza virus (d) and Candida albicans (e). Reproduced from [113] under a creative commons attribution license (CC BY). Copyright © 2021 Qin et al.

A recent study revealed the prolonged influence of iLNP on innate and adaptive immune responses for several weeks [113]. Interestingly, pre-injection of iLNP loading non-antigen mRNA or empty iLNP reduced the effect of the following iLNP vaccination injected 2 weeks later to produce antibodies and GC B cells (Figure 6(a,b)). The inhibitory effects still exist but to a lesser extent when the interval between pre-injection to vaccination increased from 2 weeks to 8 weeks. The pre-injection reduced the antigen expression efficiency in the following vaccination with mRNA iLNP, which may contribute to the inhibitory effect. A further mechanistic experiment investigated the role of innate immunity on this inhibitory effect by evaluating the effect of iLNP pre-injection on the protection from infectious diseases. In this experiment, mice receiving iLNP loading non-antigen mRNA were challenged with influenza virus and Candida Albicans 2 weeks later, wherein innate immunity determines the susceptibility to the infection (Figure 6(c)). Interestingly, the pre-injection provided a protective effect against the influenza virus (Figure 6(d)) but exacerbated the infection of Candida Albicans (Figure 6(e)). Reduced levels of neutrophils in the blood after the iLNP pre-injection may explain the results in Candida Albicans, while mechanisms involved in the influenza virus challenge experiment are unclear. Importantly, both results indicate the prolonged impact of iLNP injection on innate immune responses against following infectious diseases. By showing the inhibitory effect of iLNP on the following vaccination, this study may have clinical implications. The efficacy of COVID-19 vaccines in humans improved by prolonging the interval between prime and boost doses [114]. The inhibitory effect of the prime dose on the boost dose might explain this clinical result.

Collectively, this section provides examples of excessive adjuvanticity hampering vaccination effects, whereas adjuvanticity is an essential component of mRNA vaccines, as described in the earlier sections. The intensity, signalling pathway, and kinetics of immunostimulation may partially explain the Jekyll and Hyde nature of adjuvanticity, although further mechanistic studies are needed to understand and control the outcome of adjuvanticity.

## Delivery

### Overview of delivery

**Figure 7.**
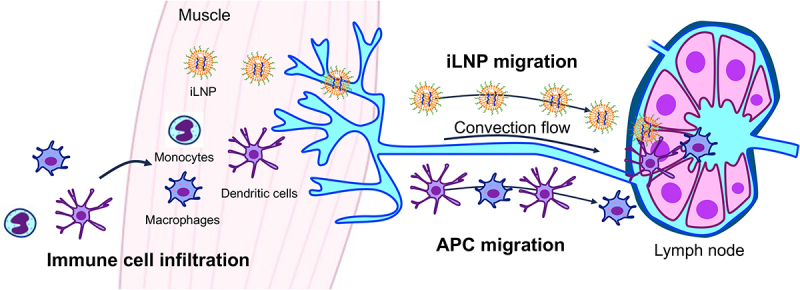
Plausible mechanisms of iLNP mRNA vaccines after their intramuscular injection. In one pathway, iLNPs migrate to the lymph nodes to provide antigen protein expression in APCs in the lymph nodes. In the other, immune cells infiltrate into the injection sites and migrate to the lymph nodes after taking up antigens.

Efficient vaccination requires antigen-expressing APCs in the lymphoid organs, including the lymph nodes and spleen, to cooperate with T cells and B cells for adaptive immunity induction [115]. mRNA vaccines have two major pathways to achieve antigen expression in APCs in lymphoid organs (Figure 7). In one pathway, after mRNA vaccine administration in non-lymphoid tissues, residual APCs take up antigen mRNA at the injection site and then migrate to the lymphoid tissues via lymphatic vessels. These processes take hours to days. In tissues possessing few APCs, this pathway requires immune cell infiltration to the injection site triggered by the innate immune responses to mRNA vaccines. Delivering mRNA vaccines to tissues rich in APCs, such as dermal tissues, is another strategy to utilize this pathway. In the other pathway, vaccine nanoparticles travel through lymphatic vessels and deliver mRNA directly to APCs in the lymphoid tissues, which takes minutes to hours. Notably, lymphoid vessels have a convection flow to facilitate this process. This pathway requires nanoparticles with size and surface properties suitable for lymphatic transport. Moreover, targeting ligands can assist the vaccine accumulation and retention in the lymphoid tissues and deliver vaccines to specific types of immune cells. Vaccine retention in the lymphoid organs is essential from the safety standpoint to avoid vaccine distribution to non-lymphoid organs, ectopic antigen expression, and innate immune activation. The following sections provide delivery viewpoints in mRNA vaccination, focusing on delivery routes and nanoparticle designs (Table 2). Each section starts with a general description of each vaccination delivery route before focusing on mRNA vaccination.

**Table 2.** Delivery strategies in mRNA vaccines.

Category	Technologies	Ref.
Lymph node targeting	Screening of iLNPs based on protein expression in the lymph nodes	[116]
	Polyplex with effective accumulation in the lymph nodes	[83]
	Lipopolyplex with effective accumulation in the lymph nodes and spleen but not in the liver.	[10]
Intradermal delivery	Protamine/mRNA complexes	[80,117,118]
	Naked mRNA with a GM-CSF adjuvant	[119]
	Dose sparing of approved COVID-19 mRNA vaccines	[64,120,121]
	Jet injection of protamine/mRNA complexes	[122]
	Microneedle comprised of dissolvable polymer matrix containing mRNA iLNP	[123]
Systemic delivery	Anionic lipoplex targeting the spleen	[8,9,62,124]
	Amphiphilic carbon dot targeting the spleen	[125]
	Lipopolyplex with (tri-)mannose ligands targeting splenic dendritic cells	[126,127]
	iLNP coated with sialic acids targeting splenic dendritic cells	[128]
Mucosal delivery	Intranasal delivery of lipid-based carriers	[129]
	Intranasal delivery of lipopolyplexes	[130]
	Intranasal delivery of iLNP with substitution of helper lipids with cationioc lipids	[76]
	Intranasal delivery of polyplex from cyclodextrin-PEI	[131,132]
	Delivery of PEGylated delivery to the lung	[133]

### Delivery to non-lymphoid tissues

Most vaccines available in clinics are injected into the muscle or the subcutaneous tissue, although these tissues are scarce in APCs [134]. Accordingly, APCs infiltrating the injection site need to take up vaccines and migrate into the proximal draining lymph nodes towards chemokine gradients to facilitate the orchestrated activation of the adaptive immune response. Alternatively, the vaccines may directly travel to these lymph nodes via the lymphatic vessels. Compared to the s.c. route, the i.m. route generally has a lower risk of adverse events at the injection site and induces a more robust immune response [135]. Muscle tissue is typically more abundant in vascular and lymphatic circulation, allowing efficient transport of vaccines to the lymph nodes [136]. This might explain the advantage of the i.m. route over the s.c. route in vaccination. In contrast, vaccines administered subcutaneously tend to stay at the injection sites for prolonged periods, presumably because of limited vascularization and low capability of substance exchange in the subcutaneous fat tissue. Accordingly, the s.c. route in vaccination relies on APC migration into the lymph nodes rather than vaccine migration.

Before mRNA vaccines attracted much attention, extensive research in other fields addressed how the physicochemical characteristics of vaccine nanoparticles determine their fate after interstitial administration. Particularly, their size and surface properties, such as electric charge and hydrophilicity, are determinants of their lymph node translocation [137,138]. Nanoparticle sizes between 10 and 100 nm, more specifically 20–50 nm, are suitable for traversing the interstitium and entering and travelling through lymphatic vessels [139]. However, nanoparticles with a size of 100–200 nm exhibit poor migration into lymph nodes because approximately 100 nm-sized water channels constrain their diffusion and convection through the interstitium [140]. Nanoparticles over 200 nm in size stay at the injection site and entirely rely on uptake by APCs and APC migration to the lymph nodes to provide vaccination effects [141]. In contrast, nanoparticles smaller than 10 nm preferentially enter blood vessels rather than lymphatic vessels because the size allows penetration through the blood capillary wall [140,142]. Moreover, flow in blood capillaries is approximately 100–500 times higher than lymphatic flow, facilitating the clearance of nanoparticles [143].

Regarding the surface properties of nanoparticles, positively charged nanoparticles are entrapped by negatively charged cell membranes and extracellular matrices at the injection site [144,145]. In contrast, negatively or uncharged nanoparticles, lacking such local interactions, can travel through the lymph vessels to the lymph nodes, exhibiting a suitable property in vaccination [146]. Coating the nanoparticle surface with hydrophilic stealth materials, including polyethylene glycol (PEG), may further enhance the migration efficiency of nanoparticles to the lymph nodes, as such surfaces minimally interact with interstitial matrices [147–149]. Intriguingly, nanoparticle PEGylation enables the translocation of over 100 nm-sized nanoparticles, such as 120 nm-sized anionic liposome [150] and 250 nm-sized cationic liposomes [151], presumably because PEGylation may improve the colloidal stability of nanoparticles and reduce their non-specific adsorption to the interstitial environment at the injection site [152]. Meanwhile, PEG density on the nanoparticle surface needs optimization. For instance, cationic liposomes incorporated with 1 mol% of DSPE-PEG2000 exhibited prolonged retention in the lymph nodes and enhanced uptake by APCs than those with 5 mol% of DSPE-PEG2000, while both liposomes efficiently migrated to the lymph nodes [151]. Another study also revealed the importance of PEG density optimization to maximize vaccination efficacy [138]. Ligands for APC targeting, such as mannose derivatives [153] and antibodies against DC40, CD11c, and DEC-205 can also enhance the delivery efficiency to APCs [154]. However, these approaches increase the cost and complexity of manufacturing [137]. Considering the high phagocytosis activity of APCs and their abundance in lymph nodes [155], tuning the physicochemical properties of the nanoparticles could be sufficient once they reach APCs in lymph nodes.

These studies on nanoparticle vaccines provide a basis for mRNA vaccine development. According to a previous mRNA study, the direct injection of naked mRNA vaccines into the lymph nodes resulted in robust CTL responses, whereas naked mRNA vaccines failed to induce detectable vaccination effects in other delivery routes, including i.d., s.c., and near-nodal injection [156]. The intranodal naked mRNA injection also showed promising outcomes in a clinical trial of cancer vaccines targeting neo-antigens [157]. These studies highlight the importance of obtaining antigen expression in the lymph nodes. However, the intranodal injection requires an arduous procedure under the guidance of ultrasound imaging, hampering widespread clinical application. In contrast, local injections performed in ordinary medical care, such as s.c. and i.m. injections, target non-lymphoid tissues. Therefore, mRNA vaccines should possess functionalities to induce antigen expression from APCs in the lymphoid tissues after delivery to the non-lymphoid tissues. Facilitating the lymph node migration of mRNA vaccines and APCs are two major pathways for this purpose (Figure 7), while these pathways are often indistinguishable.

Intriguingly, mRNA iLNPs efficiently produce antigen-expressing APCs at the draining lymph nodes after i.m. injection, which may contribute to the high efficacy of approved COVID-19 mRNA vaccines. A previous study tracked the radio-labelled iLNP in NHPs by positron emission tomography-computed tomography [158]. Within 4 h after i.m. injection, iLNP provides strong signals at the lymph nodes with a distance as far as 9.2 cm from the injection site. Other studies provided further insights into the processes after local delivery of mRNA iLNPs at cellular levels by observing mRNA distribution and reporter protein expression in small and large animals after i.m. injection of iLNP [11,60]. iLNP induced immune cell infiltration into the injection site and provided reporter protein expression in infiltrated immune cells, as well as residual fibroblasts and adipocytes. The reporter protein expression was efficiently observed in the draining lymph node but not in non-draining lymph nodes 8 h after the injection. The expression levels gradually decreased but were still above the detection limit 7 d after the injection. Among various cell types in the lymph nodes, macrophages in subcapsular and medullary sinuses exhibited robust reporter expression, while the expression is less efficient in dendritic cells and monocytes and almost undetectable in T and B cells. The macrophages may function as APCs to trigger robust adaptive immunity by interacting with T and B cells. Notably, human draining lymph nodes also exhibited efficient accumulation of vaccine mRNA and antigen protein expression after i.m. vaccination [12,159]. Although the studies above do not precisely discriminate the two pathways of obtaining antigen expression at the lymph nodes, *i.e*., migration of iLNPs and APCs (Figure 7), fast accumulation of iLNP in distant lymph nodes implies the contribution of iLNP migration. In addition, iLNP induced robust protein expression in systemic organs, including the liver and spleen [10], presumably via iLNP migration rather than APC migration, highlighting the potential of iLNP to travel outside the injection site.

Several studies have attempted to further distinguish the contribution of these two pathways. For this purpose, a recent study prepared a system with high antigen protein expression efficiency at the injection site and low efficiency at the draining lymph nodes [160]. A mixture of mRNA and empty iLNP provided comparable levels of protein expression at the injection site but much lower levels in the draining lymph node compared to iLNP encapsulating mRNA after i.m. injection. Notably, the mixture of mRNA and empty iLNP induced humoral and cellular immunity less efficiently than iLNP loading mRNA. These results indicate the significant contribution of antigen expression in the lymph nodes in vaccination. Although this study cannot precisely discriminate between the migration of iLNPs and APCs, it is reasonable to assume that iLNP encapsulating mRNA may deliver antigen mRNA into the lymph nodes more efficiently than the mixture of mRNA and empty iLNP. Thus, the results suggest the substantial role of iLNP migration to the draining lymph nodes in vaccination. Considering the size effect of nanoparticles on their lymph node migration efficiency, the sizes of mRNA iLNPs may also influence their vaccination outcomes. In this context, a previous study retrospectively studied this issue using 135 iLNP lots in mice, showing a clear correlation between antibody production efficiency and iLNP sizes [161]. This result supports the involvement of iLNP migration to the lymph nodes in vaccination. Meanwhile, the correlation became less evident in NHPs, which may reflect the difference in lymphatic system anatomy between mice and NHPs.

**Figure 8.**
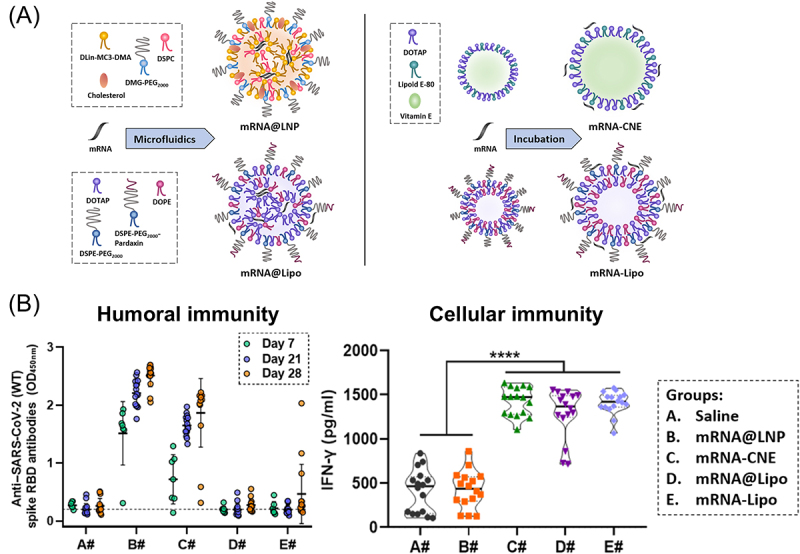
Vaccination effects of different mRNA formulations. (a) mRNA vaccine formulations. (b) Humoral and cellular immunity induction of each vaccine formulation targeting the receptor-binding domain (RBD) of the SARS-CoV-2 spike protein. Reproduced from [162] under a creative commons attribution license (CC BY-NC). Copyright © 2022 shi et al.

Another study compared the vaccination effects of iLNP, cationic nanoemulsion, and cationic liposome, revealing that the target cell types of each formulation influence the induction efficiency of humoral and cellular immunity (Figure 8(a)) [162]. After i.m. injection, iLNP produced higher levels of antibody production yet lower levels of cellular immunity than the other formulations (Figure 8(b)). In a reporter assay, the cationic liposome induced protein expression in the draining lymph nodes in mice more efficiently than the others. In a cultured cell experiment, iLNP showed a preference for myocytes over dendritic cells in terms of protein expression. Conversely, cationic nanoemulsion and liposomes demonstrated efficient protein expression in dendritic cells. Based on these results, the authors hypothesized that the efficient accumulation of cationic liposomes in the lymph nodes and efficient mRNA delivery to the dendritic cells contribute to the robust induction of cellular immunity. In contrast, iLNP might provide protein expression mainly in the myocytes, followed by APCs taking up the antigens released from the myocytes. This process may preferably induce humoral immunity. Importantly, antigen protein expression in non-APCs contributes to vaccination outcomes in several modes of action [163]. When mRNA encodes secreted antigens, APCs can take up antigens secreted from non-APCs. In the study of plasmid DNA (pDNA) vaccines, pDNA encoding secreted OVA produced higher levels of anti-OVA antibody than that encoding membrane-bound or cytoplasmic OVA after i.m. and i.d. injection in a naked form, showing the benefit of antigen secretion [164]. Meanwhile, in mRNA iLNP vaccines, mRNA-encoding cell-associated and secreted haemagglutinin (HA) showed comparable anti-HA antibody production [165], suggesting a marginal role of antigen secretion in this setting. In another mode of action, cell debris of non-APCs can also provide antigens to APCs when antigen-expressing non-APCs are damaged after vaccination [163].

Several studies indicate that mRNA vaccines do not require migration to lymphoid tissues for effective vaccinations. A recent study performed i.d. injection of naked saRNA that preferably replicates in the dermal temperature range (30–35°C) but becomes inactivated at body temperature or above 37°C [166]. The distribution of naked RNA beyond the injection site is implausible because naked mRNA may be degraded during migration to other organs. Additionally, non-optimal temperatures in the other tissues restrict saRNA replication only in the skin. This vaccine formulation induced robust cellular immunity even with antigen expression only in the skin. Another study showed robust induction of humoral immunity by cationic nanoemulsion-based saRNA vaccines even with minimal distribution to the lymph nodes or other organs [167]. In the saRNA vaccine, saRNA was attached to the surface of the nanoparticles, in contrast to iLNP loading mRNA inside the lipid layer. The study compared biodistribution and vaccination effects between cationic nanoemulsion and iLNP. After i.m. injection in mice, the nanoemulsion restricted protein expression mostly to the injected muscle, while iLNP provided strong protein expression in the draining lymph nodes and liver. However, the nanoemulsion induced more robust humoral immune responses than iLNP, indicating that the migration to the lymph nodes is indispensable in vaccination. Notably, such a localized distribution of the nanoemulsion allowed for avoiding systemic inflammatory responses, while iLNP induced systemic cytokine release and weight loss.

Notably, a recent study revealed substantial systemic distribution of mRNA iLNP in NHPs, with injected mRNA detected in the spleen, liver, and plasma after i.m. injection [11]. Such systemic distribution might cause safety problems in humans, including rare cases of autoimmune hepatitis and myocarditis. A human study of autoimmune hepatitis revealed the existence of CD8^+^ T cells targeting the spike protein in the liver [116]. This result suggests that the T cells may attack hepatocytes expressing the spike protein after mRNA vaccination. Regarding myocarditis, an investigation of mRNA distribution in patients who died after mRNA vaccination revealed the presence of vaccine-derived mRNA in the myocardium in several patients [12]. Interestingly, the sections of the heart in such patients visualized the evidence of heart injury with macrophage infiltration, while such changes were much less frequent in patients without vaccine mRNA detection in the heart. These results imply a causal relationship between myocarditis and iLNP distribution to the heart.

Although these studies highlight potential safety risks of the systemic distribution of mRNA vaccines, mRNA vaccines preferably travel beyond the injection sites to the lymphoid organs. Therefore, researchers attempt to develop mRNA vaccine formulations that migrate to the draining lymph nodes without systemic leakage [55]. For example, a previous study screened iLNP formulations based on the protein expression levels in the draining lymph nodes, using a library of ionizable lipids and iLNP with different mixture ratios of four lipid components [168]. Compared to the iLNP used in BNT162b, a clinically approved vaccine, the optimal iLNP provided enhanced protein expression in the lymph nodes and minimized expression in the liver or other systemic organs. Another study provides mechanistic insight by investigating the size effect of iLNPs using iLNP with sizes of approximately 100 nm (small), 180 nm (medium), and 330 nm (large) [169]. After i.m. injection, small and medium iLNPs leaked from the injection site to accumulate and provide strong protein expression in the liver, whereas a large iLNP showed minimal leakage from the injection site.

**Figure 9.**
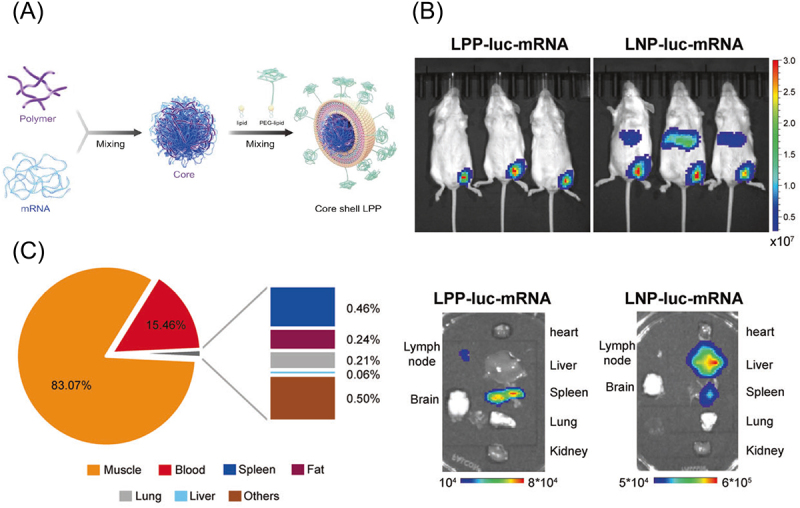
Biodistribution of a lipopolyplex. (a) Preparation of the lipopolyplex. (b) Luciferase expression distribution after intramuscular injection of the lipopolyplex and iLNP loading *luciferase* mRNA. (c) mRNA distribution evaluated by qPCR after the lipopolyplex injection. A pie chart showed the cumulative mRNA copies in each organ. Reproduced from [10] under a creative commons attribution license (CC BY). Copyright © 2021 Yang et al.

Using delivery systems other than iLNPs may also mitigate the systemic spillage of mRNA vaccines, as iLNPs have an active mechanism in liver accumulation. Apolipoprotein E (ApoE) binding to iLNP in the blood circulation facilitates its uptake by hepatocytes via low-density lipoprotein receptors in the liver [170,171]. Without such an active mechanism, some lipopolyplexes and polyplexes avoided accumulation in the liver but allowed efficient delivery to lymphoid organs, enabling safe and effective vaccination [10,83]. A study of lipopolyplexes compared its distribution profile with that of iLNP using *luciferase* mRNA after i.m. injection (Figure 9(a)). The lipopolyplexes provided efficient luciferase expression in the lymphoid organs, including the lymph nodes and spleen, with minimal expression in the liver, while the liver is the major organ expressing luciferase after iLNP injection (Figure 9(b)). In quantitative PCR evaluation after lipopolyplex injection, 83% of mRNA stayed in the injected muscle, with the remaining mRNA mostly distributed in the blood and only 0.06% distributed in the liver (Figure 9(c)). These results showed a favourable distribution profile of the lipopolyplexes. This lipopolyplex induced robust vaccination against SARS-CoV-2 in mice and NHPs, showing protective effects against infection. The vaccines exhibited an acceptable safety profile with a transient increase in systemic cytokine release and white blood cells.

### Intradermal delivery

The skin is a highly immunogenic tissue compared to muscle and subcutaneous tissues, offering an attractive site for vaccine administration [172]. Immune-related cells in the epidermis include keratinocytes, APCs, such as Langerhans cells, dermal dendritic cells, and macrophages, along with specific subsets of T cells, working together to trigger both innate and adaptive immunity. Keratinocytes, which make up 80% of all epidermal cells, express a range of pattern recognition receptors and contribute to developing an immunogenic environment by secreting proinflammatory cytokines (IL-1α, IL-1β, IFN-α, IFN-β, and G-CSF) and cytokines that facilitate the migration and differentiation of Langerhans cells (IL-6, IL-10, IL-18, and TNF-α) [117]. Langerhans cells and dermal dendritic cells are proficient at capturing antigens and migrating to the draining lymph nodes for antigen presentation, thereby activating the adaptive immune response. The dermal environment, abundant in blood and lymphatic vessels, attracts immune cells and facilitates their entry into the lymphatic flow in response to pro-inflammatory stimuli, allowing efficient vaccine delivery to the lymph nodes.

These favourable features of dermal tissues as an immune organ have prompted the development of i.d. mRNA vaccines for many years. Early animal studies have demonstrated the potential of i.d. protamine/mRNA vaccines against infectious diseases [118] and cancers [80]. The i.d. protamine/mRNA vaccines also provided detectable antigen-specific T cell responses in a human clinical trial of cancer vaccines [122]. Moreover, i.d. jet injection of the protamine/mRNA vaccines targeting rabies virus glycoprotein induced robust neutralizing antibody and CD4^+^ T cell responses in humans without serious safety concerns [119]. The i.d. administration route has also demonstrated its clinical potential even in a naked mRNA vaccine. Using granulocyte-macrophage colony-stimulating factor (GM-CSF) as an adjuvant, the naked mRNA vaccine targeting several tumour-associated antigens slowed the progression of renal cancer with the induction of both CD4^+^ and CD8^+^ T cells responsive to the antigens in some patients in a clinical trial [64]. More recently, BNT162b2 and mRNA-1273, iLNP-based COVID-19 vaccines clinically approved for i.m. administration, were tested for their potential after i.d. injection in clinical trials. i.d. injection of 10 μg or 20 μg of mRNA-1273 produced spike-specific antibodies at levels almost comparable to those induced by i.m. injection of 100 μg mRNA, suggesting the potential of i.d. administration for dose sparing [120]. The clinical studies of BNT162b2 evaluated the vaccination outcomes as a booster dose in the vaccinees of inactivated vaccines. In the studies, i.d. injection of one-fifth of the dose and i.m. injection of the full dose induced almost comparable humoral and cellular immune responses against the spike protein [121,173]. Notably, i.d. vaccination of one-fifth dose mitigated systemic reactogenicity compared with that observed after injection of BNT162b2 at a full dose.

**Figure 10.**
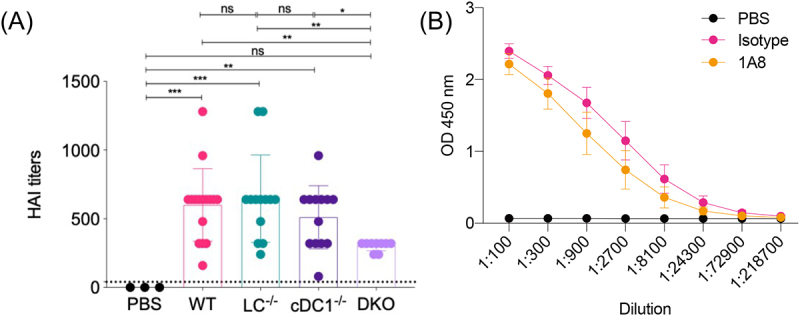
Roles of Langerhans cells (LCs) and conventional dendritic cell 1 (cDC1) in intradermal mRNA vaccination. mRNA expressing HA was intradermally injected using iLNPs. (A) Inhibition titer in wild-type (WT) mice or knockout mice lacking one or both of LCs and cDC1. (B) Antibody levels in WT mice injected with neutrophil-depleting antibodies (1A8) before vaccination. Reproduced from [59] under a creative commons attribution license (CC BY). Copyright © 2022 Ndeupen et al.

A mechanistic study undertook a detailed comparison between i.d. and i.m. mRNA vaccination using NHPs [60]. In iLNP-based mRNA vaccines targeting the influenza virus, the i.d. route outperformed the i.m. route in producing antigen-specific antibodies and CD4^+^ memory T cells. Both i.d. and i.m. iLNP administrations resulted in the infiltration of circulating neutrophils, monocytes, and dendritic cells into the injection sites. Notably, i.d. administration stimulated the proliferation of CD209^+^ residual dermal APCs. Intriguingly, after i.d. delivery of fluorescence-labelled iLNP loading mRNA expressing reporter proteins, CD1a^+^ and CD209^+^ dermal APCs efficiently took up iLNP, provided reporter protein expression for up to 9 days, became activated to express CD80, and migrated to the draining lymph nodes. These dermal APCs might enhance the vaccination effects after i.d. delivery. A more recent study evaluated the contribution of Langerhans cells and conventional type 1 dendritic cells (cDC1) using knockout mice lacking these cell types after vaccination with iLNP loading saRNA [59]. Mice lacking both cell types exhibited impaired induction of vaccine-specific antibodies in serum and Tfh and GC B cell responses in the draining lymph nodes (Figure 10(a)). Interestingly, those lacking either one cell type showed no impairment of vaccination effects, indicating redundant roles of Langerhans cells and cDC1s in mRNA vaccinations. The same study also explored the role of infiltrating neutrophils in vaccination by evaluating the vaccination effects with or without neutrophil depletion. Neutrophil depletion did not significantly decrease Tfh and GC B cell responses and antibody production (Figure 10(b)), indicating a minor contribution of infiltrating neutrophils in i.d. mRNA vaccination.

As shown in Figure 7, mRNA vaccines have two mechanisms to obtain antigen expression from APCs in the lymph nodes: migration of tissue residual APCs to the lymph nodes and that of mRNA vaccine nanoparticles. In this context, the i.d. vaccination studies in this section indicate the large contribution of the former mechanism by demonstrating the critical role of dermal tissue residual APCs. Meanwhile, not all mRNA vaccines benefit from i.d. administration routes. For example, previous reports of saRNA vaccines showed that the i.d. route provided a comparable or less efficient vaccination effect than the i.m. route [174,175]. Other factors, including the efficiency of antigen protein expression, saRNA biodistribution, and adjuvanticity, might be determinants of vaccination outcomes in these settings.

The i.d. vaccination route faces technical difficulties and inaccuracy of injection in clinical settings. This issue prompted the development of new injection methods and devices [176,177]. Among them, a jet injector was used in a clinical trial of the rabies virus [119]. Intriguingly, a jet injection of protamine/mRNA polyplex, but not a needle injection of the polyplex, induced detectable antibody production in humans. This result revealed an additional benefit of jet injection to improve vaccination effects, presumably by enhancing the delivery efficiency of the polyplexes. Microneedles provide another promising option for i.d. vaccination. For example, naked mRNA and lipid mRNA carriers provided efficient reporter protein expression in porcine skin after injection using a hollow microneedle, which allows liquid injection from a pore at the tip of each needle [123]. A more recent study has developed microneedles comprised of a dissolvable polymer matrix containing mRNA iLNP [178]. The microneedle is dissolved in 30 min after insertion into the skin to release iLNP. This system exhibited robust vaccination effects against SARS-CoV-2 at levels comparable to i.m. injection of the same iLNP. Moreover, this system is tolerable for 6-month storage at room temperature, showing no significant decrease in protein expression efficiency after storage.

### Systemic delivery

Systemic administration via intravenous injection offers an effective route to reach organs or tissues throughout the body. Its low invasiveness, technical simplicity, and universal applicability are also attractive compared to other injection routes. Notably, systemic administration of recombinant protein cancer vaccines has demonstrated high therapeutic efficacy against melanoma [62] and colorectal cancers [179]. Among the target organs, the spleen and lymph nodes play pivotal roles in vaccination as secondary lymphoid organs rich in immune cells, including T cells, B cells, and dendritic cells [148,180]. In particular, the predominant targets of vaccines are the spleen in intravenous injection and the lymph nodes in local administration.

Many years of research in fields other than mRNA vaccines have tackled a formidable challenge in controlling the biodistribution of systemically administered nanoparticles. The physicochemical properties of nanoparticles determine their biodistribution after systemic administration. For instance, nanoparticles should have a diameter larger than 6 nm or a molecular weight larger than 50 kDa to avoid rapid clearance via kidney glomerular filtration [181]. Nanoparticles sized between 10 and 200 nm preferentially accumulate in the liver, a major scavenging organ [182]. This size range aligns with the pore size in liver sinusoidal endothelial cells (50–200 nm) [183] and the size-dependent phagocytic activity of Kupfer cells [184]. In addition, slow blood flow in the liver sinusoid facilitates the capturing of nanoparticles to the sinusoidal wall [185], and a large weight of the liver is also a cause of nanoparticle accumulation in the liver. Vaccine nanoparticles should avoid these clearance and scavenging mechanisms for improving spleen targeting. Coating the nanoparticle surface with biocompatible hydrophilic polymers, such as PEG or zwitterionic polymers, can effectively reduce the liver uptake of nanoparticles [152,186]. PEGylation of liver sinusoid endothelial walls, instead of nanoparticles, can also suppress the liver accumulation of nanoparticles [187]. The spleen physically captures nanoparticles larger than 200 nm via the endothelial slit of the splenic sinuses, which filters blood components [188], while micrometre-sized aggregates are subject to entrapment by lung microvasculature [189]. Moreover, altering the surface charge of nanoparticles from positive to negative [171] or modifying them with hydrophilic polymers can significantly improve their spleen selectivity [124]. Collectively, sizes slightly larger than 200 nm, anionic surface, and hydrophilic stealth coating may be suitable properties for spleen targeting, while targeting ligands of splenic APCs can further enhance the splenic accumulation of the nanoparticles.

**Figure 11.**
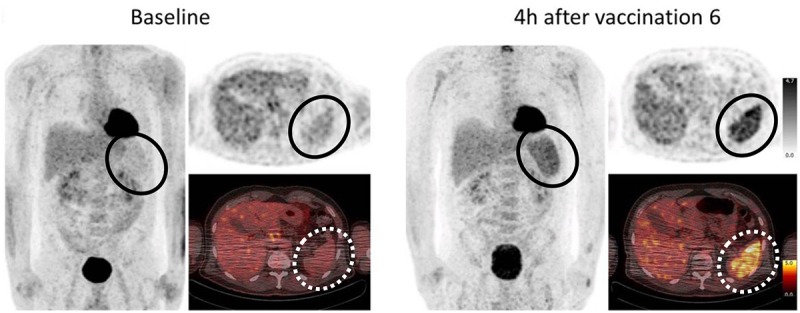
Targeting the human spleen using lipoplexes in systemic administration. ^18^F-2-deoxy-2-D-glucose positron emission tomography in a human after intravenous vaccination using anionic lipoplex. Spleens are encircled. Reproduced from [190] under a creative commons attribution license (CC BY). Copyright © 2018 pektor et al.

In the mRNA vaccine field, a pioneering study has demonstrated the utility of systemic mRNA vaccination against cancer in animal experiments and a human clinical trial [62]. In the study, lipoplexes with different surface charges, prepared by varying the mixture ratio of mRNA and cationic liposome, were screened based on their mRNA delivery efficiency in the spleen after systemic injection into mice. In a reporter assay, anionic lipoplexes, prepared with an excess charge ratio of mRNA to cationic liposome, efficiently delivered mRNA to the CD11c^+^ dendritic cells in the spleen, lymph nodes, and bone marrow, providing protein expression. This system demonstrated efficient treatment effects in several mouse models of cancers and induced detectable antigen-specific T cells in humans. Subsequent clinical trials demonstrated robust anti-tumour effects of this system by targeting tumour-associated antigens [8] and neo-antigens [9]. Fluoro-2-deoxy-2-D-glucose positron emission tomography (FDG-PET) in a human showed an increase in glucose uptake selectively in the spleen after the delivery of this anionic lipoplex (Figure 11) [190], suggesting the successful spleen targeting of this system also in humans.

**Figure 12.**
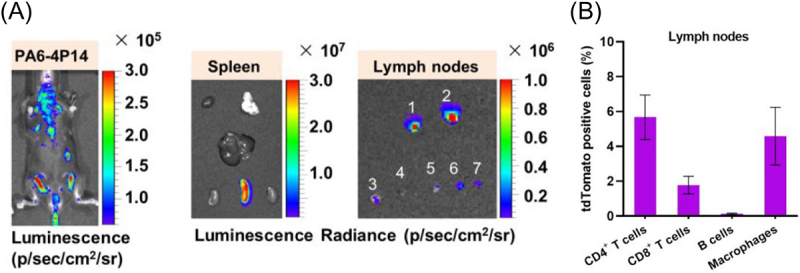
Targeting lymphoid tissues using polyplexes in mice. Reporter mRNA encoding luciferase (a) and tdTomato (b) was systematically injected. (a) Distribution of luciferase expression. (b) Cell types expressing tdTomato in the lymph nodes. Reprinted with permission from [125]. Copyright © 2021 American Chemical Society.

This clinical success is prompting the development of mRNA delivery systems targeting the spleen. For example, adding anionic lipids to mRNA iLNP redirected the iLNP from the liver to the spleen, while iLNP has intrinsic tropism for the liver [191]. A following mechanistic study suggests that the addition of anionic lipids changed the components of the protein corona attached to the iLNP in the blood circulation, which may determine the tissue tropism of the iLNPs [171]. Similarly, polyplexes from reporter mRNA and a polycation provide reporter protein expression selectively in the spleen and lymph nodes after intravenous injection in mice (Figure 12(a)) [125]. The polycation possessed a zwitterionic structure with negatively charged phosphate groups added to cationic side chains. In the lymph nodes, CD4^+^ T cells and macrophages efficiently expressed the reporter proteins (Figure 12(b)). Although anionic nanoparticles are frequently used for targeting the spleen in mRNA vaccination [62,110], a cationic nanoparticle, called an amphiphilic carbon dot, also exhibited selective mRNA delivery to the spleen [126]. The carbon dot was prepared by conjugating hydrophobic alkyl chains to polyamine-based nanoparticles. Two formulations of the carbon dot selected by *in vitro* screening were evaluated for their *in vivo* functionalities after systemic injection. One of them provided selective mRNA delivery to the spleen, inducing efficient anti-tumour activity in cancer vaccines. Collectively, these systems leverage the physicochemical properties of nanoparticles for spleen targeting. However, the precise mechanisms of the targeting remain unclear in most of the systems, and elucidation of the mechanisms will enable the rational designing of spleen-targeting systems in the future.

Along with this strategy relying on physicochemical properties, ligand-based systems are pursued as a strategy to realize precise targeting at cellular levels. A pioneering study attached mannose ligands onto lipopolyplexes, which increased mRNA delivery efficiency to splenic dendritic cells by 4-fold [127]. In the application to cancer vaccines targeting tumour-associated antigens, the lipopolyplexes showed an effective antitumor effect. However, the lipopolyplexes induced protein expression in organs other than the spleen, including the liver. To improve the selectivity for splenic dendritic cells, a subsequent study used tri-mannose instead of mono-mannose as a ligand [128]. As a result, the tri-mannose-lipopolyplexes delivered mRNA specifically to the spleen with minimal protein expression from the delivered mRNA in other organs. Furthermore, the protein expression in the spleen was drastically reduced after depleting CD11c^+^ APCs in mice, showing high specificity of this system in the splenic APCs. Interestingly, this system-induced CTL responses in a manner independent of type I IFN and thus retained the CTL activity even with 1 mΨ modification. Consequently, the lipopolyplexes loading 1 mΨ-modified mRNA offered effective cancer vaccines with minimal induction of systemic cytokine release. Another group used sialic acid to target sialic acid-binding immunoglobulin-like lectin-1 (Siglec-1) expressed on the surface of dendritic cells as a ligand of iLNP [192]. Interestingly, the introduction of sialic acid to iLNP drastically increased the endosomal escape efficiency. As a result, the iLNP allowed for efficient mRNA delivery to the splenic dendritic cells after systemic administration, thereby potentiating anti-cancer vaccines.

### Mucosal delivery

Mucosal vaccination can avoid invasive injection procedures and trigger robust local and systemic immune responses in mucosal-associated lymphoid tissues (MALT) [193]. In particular, the mucosa in the upper and lower respiratory tract, including the lung, is an attractive site for vaccines targeting infectious respiratory diseases [194]. Intranasal (i.n.) administration or inhalation of live attenuated and adenovirus vector mucosal vaccines against COVID-19 exhibited promising outcomes in clinical trials [195]. In mucosal immunity, IgA secreted into the mucus plays a significant role in neutralizing and eliminating foreign pathogens in their first entry sites, effectively preventing infection. Besides, i.n. vaccination efficiently induces cross-reactive antibodies, conferring broad cross-protective immunity [196,197]. This feature is attractive for preventing the infection of viruses with high mutation rates, such as RNA viruses. Effective induction of IgA secretion requires antigen delivery to APCs residing in the lymphoid tissues under the mucus-covered epithelial barrier. M cells in the mucosal epithelium mediate the sampling of antigens from the mucosal surface into the lamina propria [198]. Therefore, previous research on i.n. vaccination has attempted to facilitate the penetration of vaccines through mucus for their uptake by M cells, demonstrating the feasibility of this strategy [199]. Targeting APCs is also critical in this administration route, wherein B cells activated by APCs secrete IgA massively with the assistance of activated CD4^+^ T cells [200]. Notably, mucosae at different sites have crosstalk. Thus vaccination at a mucosal site can induce antigen-specific immunity in distant mucosal sites, facilitating the fortification of the entire mucosal system against pathogen infection [201]. In addition to mucosal IgA secretion, activated B cells provide systemic IgG secretion for whole-body immunity [202]. Furthermore, mucosal vaccination can induce cytotoxic CD8^+^ T cells to kill infected cells or antigen-expressing cells directly, motivating the application to cancer vaccines [129].

Despite these advantages of mucosal vaccination, the application of mRNA vaccines remains challenging, with very few reports addressing this topic. In a previous study on vaccines against the influenza virus and SARS-CoV-2, i.n. administration of saRNA iLNP resulted in more than a 10-fold lower antibody titre and less efficient cellular immunity than its i.m. injection [91]. Mechanistic analyses revealed higher serum cytokine levels after i.m. injection than after i.n. administration. Thus, the authors explain the reduced vaccination efficiency in i.n. route by the low adjuvanticity. A subsequent study conducted a more comprehensive comparison among i.n., i.m., and i.d. vaccination of saRNA, using 4 different vaccine formulations: solid LNP, polymeric nanoparticles, cationic LNP, and iLNP [175]. In each formulation, i.n. vaccination consistently produced less efficient humoral and cellular immunity than the other vaccination routes, even when i.n. vaccination employed a 10-fold higher mRNA dose than the other two routes. A luciferase mRNA reporter assay revealed that luciferase expression persisted for only a few days after i.n. administration, whereas i.m. and i.d. injections provided expression for longer than 10 days. Therefore, the rapid clearance of cells introduced with saRNA via i.n. administration might explain the low immunogenicity of i.n. saRNA vaccination.

Meanwhile, several reports have presented promising outcomes in i.n. mRNA vaccination, utilizing lipid- and polymer-based systems. In the context of the lipid systems, a pioneering study employed a commercial lipid-based reagent for i.n. mRNA vaccination, inducing anti-cancer activity after prophylactic and therapeutic administration in mouse models [130]. In another study, mRNA was loaded into PEGylated cationic lipoplex after condensation using protamine [131]. This system demonstrated efficient antigen expression and immunostimulation in cultured cells, along with anti-tumour activity in mice accompanied by efficient induction of CD4^+^ and CD8^+^ T cells. In a more recent study, iLNP was utilized for i.n. vaccination after substituting zwitterionic helper lipids with cationic lipids to enhance mRNA delivery efficiency to mucosal tissues [76]. The resulting iLNP achieved efficient mRNA delivery to mucosal APCs. Ultimately, the iLNP exhibited robust systemic IgG induction at levels comparable with those observed after i.m. vaccination using the same system when loading mRNA encoding a fusion protein of the receptor-binding domain of the SARS-CoV-2 spike protein and a complement C3 protein.

In the realm of polymer systems, a series of studies has sought to overcome nasal epithelial barriers, which impede mRNA vaccines from reaching APCs. While polycations effectively break the barrier by opening tight junctions between epithelial cells, they can induce tissue damage. Consequently, these studies modified PEI with cyclodextrin to alleviate cytotoxicity by reducing charge density. Following i.n. vaccination, the resulting polyplex successfully generated IgA in the nasal and vaginal cavities, as well as IgG and antigen-specific CD8^+^ and CD4^+^ T cells [132]. According to a subsequent mechanistic study, the polyplex effectively provided protein expression in superficial cervical, deep cervical, and axillary lymph nodes and activated dendritic cells in the lymph nodes, thereby enhancing vaccination efficiency [133]. In safety analyses, the polyplex did not induce any increase in toxicity markers or cytokines in the blood, and no abnormality was observed in tissue sections of the nasal epithelia and the liver. In a more recent study, mucosal vaccination was attempted through PEGylated mRNA polyplex delivery to the lungs [203]. For pulmonary delivery of polyplexes, surface PEG density requires optimization [204]. PEG mitigates tissue damage induced by cationic polyplexes and facilitates their mucosal penetration, although a dense PEG layer on their surface hampers uptake into target cells. The vaccine study first optimized PEG density and polycation structure through screening based on delivery efficiency to the lung. The optimal polyplex provided efficient protein expression in lung APCs and epithelial cells. In the context of SARS-CoV-2 vaccines, the polyplex induced antigen-specific T cells and B cells, including B cells expressing IgA, in the draining lymph nodes. Moreover, antigen-specific tissue-resident CD8^+^ T cells and robust mucosal secretion of IgG were observed, demonstrating successful induction of mucosal immunity. Ultimately, the system effectively protected mice from viral challenge. However, the system failed to induce detectable IgA secretion, necessitating further improvement in vaccine design.

## Future perspectives

mRNA vaccines require various functionalities, including the protection of mRNA from RNase attacks, targeting of APCs and lymphoid tissues with minimal off-target distribution, and the temporal and spatial control of innate immune activation. While the stimulation of innate immunity is crucial for effective vaccinations, excessive adjuvanticity can lead to adverse effects and hinder vaccination outcomes. In the context of mRNA delivery systems, the migration of mRNA nanoparticles to lymphoid organs is advantageous for vaccination effects. However, their widespread distribution to off-target tissues, such as the liver and heart, can result in rare but severe adverse effects [12,116]. Fine-tuning these various factors in mRNA vaccines remains challenging, necessitating large-scale screening to determine optimal mRNA vaccine designs. For instance, SM-102, an ionizable lipid used in an approved COVID-19 vaccine, mRNA-1273, was selected from 30 formulations of LNPs based on antigen production capability in mice and NHPs [90]. Notably, slight changes in ionizable-lipid structure can cause substantial differences in humoral immunity induction, while ionizable lipids with a pKa between 6.6 and 6.9 tend to exhibit efficient vaccination effects. Interestingly, while five lead LNPs demonstrated comparable antibody production efficiency in mice, they resulted in large differences in antibody induction in NHPs, underscoring species-specific variations in vaccination efficacy. More detailed deciphering of the structure-function relationship may benefit future vaccine development, and for this purpose, vigorous mechanistic analyses are being conducted, as discussed in this review.

The aforementioned species discrepancy is a pivotal concern across the field of nanomedicine. For example, there exist significant variances in nanomedicine biodistribution between preclinical animal models and humans [205]. Species-specific variations in the protein corona adsorbed to the nanomedicines can influence their distribution in each species by affecting processes of overcoming several biological barriers, such as limited tissue diffusion and undesirable clearance mechanisms. Moreover, pro-inflammatory responses, posing additional barriers to mRNA vaccines, also differ among species. For instance, mice exhibited greater tolerance to anionic mRNA lipoplexes than humans, displaying minimal proinflammatory reactions upon systemic injection [61]. Mice more effectively produced anti-inflammatory IL-1ra in response to pro-inflammatory stimuli compared to humans, mitigating the systemic reactogenicity of mRNA vaccines. Addressing species differences is critical in future vaccine development.

Current mRNA vaccines require improvement, especially in reducing reactogenicity, despite their success against COVID-19. While the reactogenicity may be deemed acceptable for a few doses during a pandemic, there is a demand for mRNA vaccine formulations with milder reactogenicity for repeated boosting against COVID-19 and broader applications in other infectious diseases. Additionally, strong reactogenicity is a cause of vaccine hesitancy [206]. The debate continues whether reactogenicity and immunogenicity (vaccine effects) are separable [30]. Systemic reactogenicity is more common after the second dose of vaccines than the first dose in humans [3,4], suggesting the involvement of adaptive immunity in reactogenicity. Moreover, enhanced reactogenicity coincided with higher levels of systemic IFN-γ secretion after the second dose in humans and mice [20,29]. In a mouse study, CD4^+^ and CD8^+^ T cells were the primary producers of IFN-γ in the second dose, and the administration of an IFN-γ receptor neutralizing antibody dampened the activation of macrophages, monocytes, and dendritic cells. These findings suggest a close relationship between reactogenicity and immunogenicity. However, an IFN-γ receptor neutralizing antibody minimally affected the efficiency of humoral and cellular immunity induction in mice. Furthermore, the intensity of systemic reactogenicity showed limited correlation with the efficacy of humoral and cellular immunity induction in humans [207,208]. These results indicate the potential to separate reactogenicity and immunogenicity. Concerning adjuvanticity, elucidating innate immune signalling pathways responsible for reactogenicity and immunogenicity could benefit the development of effective vaccines with reduced reactogenicity. mRNA delivery systems that efficiently accumulate in the lymph nodes with minimal systemic leakage [10,55,168] might mitigate systemic adverse effects while maintaining effectiveness.

While this review primarily focuses on adjuvanticity and mRNA delivery systems, the combination with other strategies is essential for the future design of mRNA vaccines. RNA designs aimed at prolonging the duration of antigen expression, including saRNA and circular RNA, can also enhance the potency of mRNA vaccines. An saRNA vaccine, ARCT-154, demonstrated enhanced immunogenicity than BNT162b2 as a booster vaccination after two doses of BNT162b2 or mRNA-1273, in a phase 3 clinical trial [209]. Circular RNA encoding the receptor-binding domain of the SARS-CoV-2 spike protein induced robust immune responses in mice and NHPs, although vaccine effectiveness was almost comparable between circular RNA and linear mRNA [210]. In studies of recombinant protein vaccines, the exposure kinetics of antigens and adjuvants drastically impact vaccination outcomes, with sustained exposure leading to enhanced antibody production [211,212]. Therefore, modulating the kinetics in mRNA vaccines could improve their efficacy. To achieve this, several reports have attempted to control the release of mRNA nanoparticles from hydrogels. For example, mRNA complexed with a lipid-based commercial reagent was loaded onto a poly (2-hydroxyethyl methacrylate) (pHEMA)-based porous scaffold [213]. After subcutaneous injection, this scaffold retained mRNA for more than 3 days and enhanced protein expression efficiency compared to bolus mRNA injection. Recent research developed a hydrogel from graphene oxide (GO) and linear PEI (LPEI) loaded with antigen mRNA and TLR7 agonists [214]. The hydrogel gradually released GO-LPEI nanoparticles loaded with mRNA and the agonist over a month after injection in mice while protecting mRNA from degradation during this period. As a result, hydrogel injection improved the anti-tumour effect compared to bolus injection of the nanoparticle. The engineering of proteins encoded by mRNA also substantially impacts vaccination effectiveness. Intriguingly, engineering the spike proteins of SARS-CoV-2 to form virus-like particles in mRNA vaccines boosted neutralizing titres by more than 10-fold [215]. In cancer vaccines, accurate epitope prediction and combination with other immunotherapies are critical [7]. Optimizing adjuvanticity and delivery systems, in combination with other strategies described above, will drastically expand the potential of mRNA vaccines in the future.
